# Ten characteristics of high-quality planetary health education—Results from a qualitative study with educators, students as educators and study deans at medical schools in Germany

**DOI:** 10.3389/fpubh.2023.1143751

**Published:** 2023-04-25

**Authors:** Johanna Simon, Sandra Parisi, Katharina Wabnitz, Anne Simmenroth, Eva-Maria Schwienhorst-Stich

**Affiliations:** ^1^Department of General Practice/Family Medicine, University Hospital Würzburg, Würzburg, Germany; ^2^Centre for Planetary Health Policy (CPHP), Berlin, Germany; ^3^Teaching Clinic of the Faculty of Medicine and Institute of Medical Teaching and Medical Education Research, University Hospital Würzburg, Würzburg, Germany

**Keywords:** climate change, climate resilience, planetary health, planetary health education, medical education, transformative education, education for sustainable healthcare, eco health

## Abstract

**Aim:**

The climate and ecological crises are considered fundamental threats to human health. Healthcare workers in general and doctors in particular can contribute as change agents in mitigation and adaptation. Planetary health education (PHE) aims to harness this potential. This study explores perspectives among stakeholders involved in PHE at German medical schools on the characteristics of high-quality PHE and compares them to existing PHE frameworks.

**Methods:**

In 2021, we conducted a qualitative interview study with stakeholders from German medical schools involved in PHE. Three different groups were eligible: faculty members, medical students actively involved in PHE, and study deans of medical schools. Recruitment was performed through national PHE networks and snowball sampling. Thematic qualitative text analysis according to Kuckartz was used for the analysis. Results were systematically compared to three existing PHE frameworks.

**Results:**

A total of 20 participants (13 female) from 15 different medical schools were interviewed. Participants covered a wide range of professional backgrounds and experience in PHE education. The analysis revealed ten key themes: (1) Complexity and systems thinking, (2) inter- and transdisciplinarity, (3) ethical dimension, (4) responsibility of health professionals, (5) transformative competencies including practical skills, (6) space for reflection and resilience building, (7) special role of students, (8) need for curricular integration, (9) innovative and proven didactic methods, and (10) education as a driver of innovation. Six of our themes showed substantial overlap with existing PHE frameworks. Two of our themes were only mentioned in one of the frameworks, and two others were not explicitly mentioned. Few important elements of the frameworks did not emerge from our data.

**Conclusions:**

In the light of increased attention regarding the connections of the climate and ecological crises and health, our results can be useful for anyone working toward the integration of planetary health into medical schools' and any health professions' curricula and should be considered when designing and implementing new educational activities.

## 1. Introduction

The climate crisis has been called the most significant threat to human health in the twenty-first century (1). It is one important element of a planetary health (PH) perspective, others include biodiversity loss, global social injustice, limits to growth (2) and the risk of civilization collapse via nuclear conflict. Effects of the climate and other environmental crises on human health worldwide can already be observed (3). These health impacts vary by region. Prevalent risks in Western Europe, including Germany, include extreme weather events, such as heat waves and flooding. Moreover, a rise in disease burden through allergies and changes in occurrence of certain infectious diseases linked to the climate crisis are observed (4–6), as are negative mental health effects, especially for young people (7, 8). The interdependence of human health and wellbeing and planetary ecosystems is at the core of the emerging concept of PH, which has been defined as “the health of human civilization and the state of the natural systems on which it depends” (9). Achieving PH requires a profound transformation of all areas of human activities, for example the energy, mobility and agri-food systems. At the same time, adaptation to the already occurring (health) impacts of the changes in planetary ecosystems is required. Education can play an important role in this regard when it “includes knowledge transfer to raise awareness of certain realities, critical analysis to understand the complexities underlying these realities, and experiential exposure to connect to these realities” (10, 11).

The health (care) sector plays a specific and important role in the mitigation of and adaptation to the unfolding planetary crises: On the one hand, it contributes to the climate and ecological crises by generating 4.4% of the global greenhouse emissions and a high resource use (12). On the other hand, it has to respond to changing disease burdens caused by these crises (3). This implies that the education of health professionals needs to be adapted so that they become equipped with the knowledge and skills they need to address these health impacts. Additionally, it has been suggested that health professionals can play an important role as change agents in driving the transformative societal changes needed to mitigate the climate and ecological crises (13, 14). Health professionals belong to the most trusted of all professional groups in society (15) and the medical professional ethos demands care for individual and population health, including that of future generations (16). In Germany, the duty to maintain natural living conditions is explicitly mentioned in the medical professional code (17).

Planetary health education (PHE) aims to “equip people with the necessary knowledge, skills and values, as well as a sense of self-confidence and self-efficacy in the face of multiple environmental and social crises, in order to collectively achieve the necessary transformation of societal activities for planetary health” [own translation (18)]. Within medical education, it cannot be expected for all students to become experts for PH topics, but it is crucial to get a general understanding of the most important aspects as well as (further) develop values aligned with planetary health in their professional identity formation.

Several conceptual frameworks, detailed road maps, and principles for PHE have recently been developed. The Association of Medical Education in Europe (AMEE) has laid out in its “Consensus Statement: Planetary health and education for sustainable healthcare” (19) examples of learning activities, opportunities, and possible assessment modes as well as a road map and targets for implementing PHE. Further conceptual frameworks that define the scope and aims of PHE are the 12 “Cross-cutting principles for planetary health education” (20) and “A framework to guide planetary health education” (21).

Although the integration of PHE into the medical curriculum has been demanded repeatedly, including by medical students in Germany and globally, the integration of PHE into medical education and the monitoring of these processes remains insufficient (22–27). Like in other countries, such as the UK (28), previous studies show that that the majority of German students is not yet familiar with the PH concept, but would like to learn more about PH and consider it relevant to their studies (29). While a growing number of curricular and extracurricular educational activities is being implemented at several institutions, including nationwide lecture series (11), the experience of stakeholders involved or interested in PHE at German medical schools has, to our knowledge, not yet been explored. The opinion of the stakeholders of what characterizes high-quality PHE could inform the process of integrating PHE into medical education in Germany.

The aim of our study therefore is to explore the insights into the characteristics of high-quality PHE of a broad range of stakeholders - educators, students as educators and study deans - who are involved in PHE at German medical faculties. We also assess whether our findings are related to existing PHE frameworks.

## 2. Materials and methods

### 2.1. Study design

This qualitative interview study is a component (substudy C, see Supplementary material S1) of the mixed methods study: Planetary Health in Medical Education in Germany (PlanetMedEd). The aim of the PlanetMedEd study is to comprehensively investigate and explore the current state and diverse perspectives of the potential ways forward for PHE at German medical schools. Further components include a nationwide student survey and a systematic overview of PHE initiatives including a systematic web search. Results of these components will be published elsewhere.

### 2.2. Study population and recruitment

Three groups were eligible for participation in this study: Educators, students as educators (18 years and older), and study deans who were involved in PHE or were interested in establishing PHE at their institution. Participants were recruited *via* the contact list of the PHE working group (30) of the German Alliance on Climate Change and Health (KLUG e.V.) and through snowball sampling. Some individuals were recruited in alumni and student groups within the Master in Medical Education (MME-D) programme at Heidelberg University. In addition, results of the web search conducted as part of the PlanetMedEd study (Substudy A) were also used to identify eligible individuals.

### 2.3. Data collection and analysis

We conducted interviews between June and September 2021 *via* the Zoom^®^ video-conferencing software or *via* telephone based on a semi-structured interview guide (see Supplementary material S2). Additionally, sociodemographic data (gender identification, age in categories, academic background, and profession) as well as further professional qualifications and activities or expertise considered relevant for PHE by the interviewees were collected to allow contextualization of the qualitative findings. The transcripts were completely anonymized and contained no personal or third-person information that would allow tracing any identities. Data on sociodemographic characteristics were only collected for contextualization and are presented in aggregated categories.

**Figure 1:**
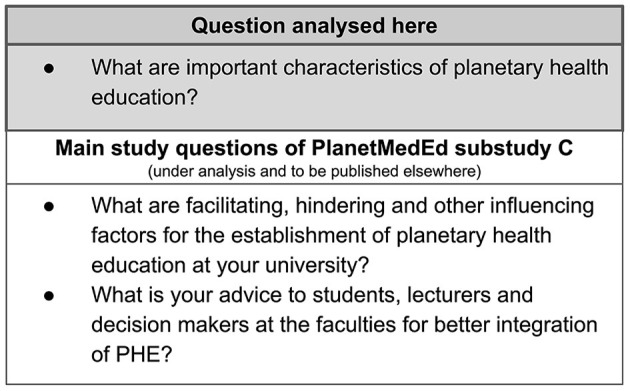
Overview of the main questions based on the semi-structured interview guide of the PlanetMedEd qualitative substudy C.

Data were collected jointly by JS (doctoral thesis student) and E-MS-S (MD, MScIH, experienced in PHE and implementation research). JS conducted 4 interviews alone. Both researchers were present in 13 interviews and took turns in conducting the interviews and note-taking. The interviews were audio-recorded and transcribed verbatim. The same two authors (E-MS-S, JS) were involved in the coding process. Thematic qualitative text analysis with a content-structuring approach according to Kuckartz was performed in MAX-QDA 2020 (31). For the current study, we deductively grouped data into main categories adapted from the main interview questions (see Figure 1), then we inductively developed sub-categories within the first main category on PHE characteristics.

The process of inductively developing sub-categories included the following steps: After familiarizing ourselves with the data, we first created sub-categories independently for three randomly selected interviews. We then compared and discussed these sub-categories. As an intermediate analysis step, case summaries were created for each interview to create a better overview of the data. A detailed system of sub-categories was then created based on all interviews and was discussed several times in the research team for completeness and consistency before it was applied to the entire text material. We performed several iterations to adjust the sub-category system.

In a second analysis step, we compared the categories developed in this way with three existing frameworks for PHE: With the “Cross-cutting principles for PHE” by Stone et al. (20), with “A Framework to guide PHE” by Guzmán et al. (21), and with the “AMEE consensus statement: Planetary health and education for sustainable healthcare” by Shaw et al. (19). For this purpose, E-MS-S, JS, and KW (MD, experienced in PHE and qualitative research) independently assessed whether our categories corresponded to elements in the central figures of each framework. Any discrepancies were resolved through discussion. In this step, we did not aim to compare the three frameworks with each other or to explore in depth how our sub-categories corresponded to the content of the entire text body supporting each framework. We rather aimed to broadly compare them in order to assess whether our data could provide new aspects that were not yet covered in the existing frameworks, or if central themes were covered in the frameworks that were not mentioned by our study participants. As guidance for a comprehensible presentation of the study conduct, the checklist presented in the Consolidated criteria for reporting qualitative research (COREQ) guideline (32) was used (see Supplementary material S3).

### 2.4. Data management and protection

The audio files were transcribed anonymously directly from the recorder and then deleted. Data storage and analysis took place on password-protected computers, in anonymized form and in accordance with the privacy policy.

### 2.5. Ethical considerations

The ethics committee of the University Hospital of Würzburg approved the conduct of the study (file number 20210312-01). All participants were provided with study information sheets and provided written informed consent.

## 3. Results

A total of 30 potential participants were identified and contacted, of which 20 individuals agreed to participate and were interviewed in 17 interviews (3 interviews were conducted jointly with 2 participants from one university). The interviews lasted between 13 and 43 min, with an average duration of 31 min.

### 3.1. Sociodemographic data of participants

Participants reported a broad range of professional and academic backgrounds. Eleven participants were faculty members, seven were students actively involved in PHE, and two were study deans. Activity in several networks such as the German Alliance on Climate Change and Health or Health for Future and other experiences were reported as relevant to their PHE expertise or interest (Table 1).

**Table 1:** Sociodemographic data of the participants.

**Characteristics**			**Number of participants**
Gender identification	Female		13
	Male		7
Age (years)	18–30		8
	31–40		5
	41–50		3
	51–60		4
Function	Students as educators	Study Progress: Clinical section/Masters degree up to internship year (“Praktisches Jahr”)	7
	Faculty		11
		Professional experience (years)	
		<1	3
		1–10	3
		11–20	
		>20	5
		Teaching experience (years)	
		< 1	3
		1–10	3
		11–20	3
		>20	2
	Study deans^a^	Professional and teaching experience: >20 years	2
**Further personal information combined for all participants**			
Academic background	Medicine with different sub-specialties, molecular biotechnology, biochemistry, public health, medical anthropology, history of medicine, nursing, physiotherapy, environmental health, tropical medicine, geography, medical education, medical informatics, research, and working experience outside of the home country		
Professional background, current occupation^b^	Preventive medicine, public health, medical education, environmental health, history of medicine, community medicine, biology, and toxicology, including various professorships in the listed areas; additionally, decision makers in non-governmental organizations		
Network activity considered relevant for PHE by interviewees^b^	German Alliance on Climate Change and Health (KLUG) and Health for Future (H4F), German branch of the International Federation of Medical student associations (bvmd), Globalization and Health Initiative (GandHI) of the bvmd, activity in various organizations working for environment, sustainability, climate mitigation, climate and health (education), or working groups in medical institutions		
Other experience and expertise considered relevant for PHE by interviewees^b^	Expertise in health education, higher education didactics, (medical) anthropology, human rights, science communication, long-term stays abroad, and long-term cooperation with partners abroad		

a Study deans were not actively involved in PHE, but supported PHE at their faculties.

b Data are presented in aggregated form for reasons of participant anonymity.

### 3.2. Characteristics of high-quality planetary health education

Ten main themes emerged (Table 2). The categories and examples of verbatim translations are listed in Table 3. The complete coding framework can be found at Supplementary material S4, verbatim supplement (VS) VS1-142 in Supplementary material S5, S6.

**Table 2:** Ten main themes.

**Ten main themes**
1. Complexity and systems thinking 2. Inter- and transdisciplinarity 3. Ethical dimension 4. Responsibility of health professionals 5. Transformative competencies, including practical focus 6. Space for reflection and resilience building 7. Special role of students 8. Need for curricular integration 9. Innovative and proven didactic methods 10. Education as a driver of innovation

**Table 3:** Ten themes, description of the themes and example verbatims.

**Theme**	**Description**	**Example verbatims**
1. Complexity and systems thinking	The complexity of planetary health topics needs to be covered adequately. This poses a challenge, and PHE shall promote systems thinking	I think it's very important that you don't cut it down to environment and health, but that you include a lot of things, how everything is connected. (VS11, P5) [...] that you don't shorten it and that you somehow accept this whole, [...] huge system, which you can't understand at all because it is so big (VS 13, P5) I think that's the attraction, but also the difficulty, that it's so wide. (VS3, P10) If you want to understand [...] then you don't have to work through all the examples and then know all the examples, but it is enough to work through a few and then you simply have an understanding of how such structures work and how you can rethink such things. (VS14, P5)
2. Inter- and transdisciplinarity	Inter- and transdisciplinary collaboration is necessary for quality PHE	You can only try to take the various diverse facets into account, and that's why it's interdisciplinary, and you just have to work with colleagues who have other perspectives and ideas and with people who have other perspectives and ideas. (VS22, P3) I thought it was great to sit down with geographers, geologists, student teachers, psychologists, and talk about teaching didactics. And I found it very enriching. And then I already had this transdisciplinary idea, which plays an enormous role in planetary health. (VS 26, P8)
3. Ethical dimension	Learners should learn to reflect from a climate justice perspective as well as to think globally and act locally. They should also reflect consumption choices	Planetary health is also related to the social reality in the world, that one does not only see it from a scientific point of view, et cetera, and from the perspective of the Global North, but it is also related to the social issues, both social inequality here with us related to climate change and also [global] climate change consequences. (VS38, P3)
4. Responsibility of health professionals	Learners should recognize and learn to use their special role as agents in the health care system	It (PHE) must clarify in any case for the students the significance they can have as physicians later in their professional life for this issue, and students must be aware that it makes an immense difference whether they perceive this responsibility or not. (VS49, P7) [...] medical students as critical citizens and physicians as responsible critical citizens in a broader sense. (VS60, P10)
5. Transformative competencies including practical skills	Transformative competencies are an aim of PHE. This includes the acquisition of practical skills	Good teaching is more than simply learning facts by heart. Good teaching also includes, in particular, conveyance of the contexts and transformation of them into knowledge for action, so that students can actually apply this knowledge in their own actions, that they can also reflect on the knowledge, that they can deduce and discuss what consequences certain actions or non-actions have. (VS63, P9)
6. Space for reflection and resilience building	Learners need space for guided and unguided reflection inside and outside the classroom; PHE also needs to incorporate resilience building for learners as they are confronted with dire future scenarios	For me, good teaching means that the students are enthusiastic, that they come up with their own ideas, that they are allowed to discover and develop their own thoughts, that they are allowed to look for solutions themselves, that they are also encouraged in the process, and that they are not given a path to follow, but that they have room to develop things. (VS74, P2) [...] so that they are also accompanied in the findings that are gained on the way and that are sometimes very sobering, frustrating or also... yes, can also move you very much. (VS 75, P2)
7. Special role of students	PHE shall be student-oriented, and their role as possible experts needs to be appreciated. This will result in a flat (or no) hierarchy between teachers and learners	To connect with the students where they are at the moment. In other words, to link to topics that they might also throw into the classroom and then to make the connection to planetary health. (VS84,P8) [...] we could always use more space in which exchange takes place, because many (students) have prior knowledge in different areas and we can also learn from each other, so not only the lecturers can teach the students something, but also the other way around. (VS88, P7)
8. Need for curricular integration	PHE needs to be integrated throughout the entire curriculum, not only as stand-alone courses or only electives	[PHE] is not something that is only added to medical studies as something completely new that would otherwise be separate from medical studies in itself and is only an add-on, an add-on, an add-on, but it is clearly something that touches the core of medical studies and maps many competencies that are important for medical training, and modern medical training extremely well and also corresponds to a great interest and a great need from the side of the students. (VS99, P3)
9. Innovative and proven didactic methods	Both innovative and evidence-based competency-oriented methods of teaching and assessment shall be used	Because PHE is now implemented de novo, I think it has to have even more sophisticated didactic concepts. I don't think a classical approach would do justice to the topic. Instead, it must be highly innovative, just like the topic itself. (VS114, P16)
10. Education as a driver of innovation	Integrating PHE in the health sciences can ideally promote sustainability in the practical work of the health care sector through a general transformation of mindsets	For me, in the end, good teaching would be a complete mindset change of the teaching staff. So, I would like to see a critical attitude of all university lecturers, be it cardiologists or dermatologists... or pneumologists, who are at the same time also [...] sensitive of the state of our planet and the cross connections to their particular field. And that they would then address this there. (VS137, P8)

#### 3.2.1. Complexity and systems thinking

Many participants stressed the importance of a solid foundation of factual knowledge that takes the complexity of the topic into account and at the same time introduces a perspective of systems thinking. The participants perceived fundamental challenges in developing a curriculum that covers the broad range of topics on PH and also discusses them in depth (VS1-3).

“Factual knowledge” (VS7) on climate and environmental changes, knowledge on environmental factors as health determinants, such as urban planning and the psychosocial environment and a historical context of the development of human societies to date were considered important (VS4). Students should be enabled to understand and classify the multiple complex interrelationships of the system and the interaction of these interrelationships with human health (VS17-19) as well as to consider patients in their entire environment (VS19). This eclipses the classical medical curriculum by far (VS15).

#### 3.2.2. Inter- and transdisciplinarity

An integral part of PHE is to bring different disciplines together and to teach and learn together (VS21, VS24-26). This concerns different professional groups and disciplines in the health sector (VS27), but also “the inclusion of other groups and members of society that have not been classically considered by us so far” (VS29). For example, interdisciplinary lectures might include lectures by geographers and space researchers (VS30).

#### 3.2.3. Ethical dimension

An ethical discussion of issues such as gender-based discrimination, racism, sexism, capitalism (VS35), and modern medical treatments and their impact on the environment (VS34) should be included. “Social justice” in terms of a “global justice perspective” (VS40, VS39) and the different regional impacts of environmental change also need to be discussed (VS38, VS41). Students should consider and reflect the principles they see as the basis of society (VS36). “Think globally and act locally” (VS46) represents an important principle here.

Other areas mentioned were individual consumption habits, including those of the students themselves, for example with regard to air travel and meat consumption, including weighing the impacts and learning how to make difficult consumption choices (VS43, VS44).

#### 3.2.4. Responsibility of health professionals

A reflection of one's own position in society as well as options for action or even agency, including the role of a change agent as a healthcare professional, should be part of PHE (VS48-50, VS53). Health professionals should develop a “sense of duty and responsibility” (VS55), and be aware of their social role-model function (VS54). This does not only mean the education of patients about PH issues, but also considers the sustainability aspects of one's own medical practice, financial investments, and mobility (VS51). This requires addressing questions of the (professional) attitude of “medical students as critical citizens” (VS59, VS60).

#### 3.2.5. Transformative competencies, including practical focus

By acquiring knowledge about possible actions and reflecting about their own options for action, students themselves can become change agents and can gain confidence in their own agency (VS61-63, VS65-67), motivating the implementation of own ideas (VS68). Excursions with practical experience can be helpful in this regard, for instance a visit of a sustainable farm (VS64).

Science competencies, including searching, finding, correctly classifying, and evaluating sources of scientific evidence is relevant in order to be able to communicate knowledge properly and also to apply it to one's own actions (VS69-71). Thereby, it can also be part of PHE to acquire and practice communicative skills (VS72) by talking to other groups of society, for example in schools and nursing homes to support transformation within communities (VS73).

#### 3.2.6. Space for reflection and resilience building

Learners need enough space for guided and unguided reflection inside and outside the classroom, to reflect and discuss freely and to share frustrating experiences (VS74-76). This is important to question one's own behavior and values, to promote “systemic thinking, critical thinking” to “broaden the horizon” (VS80), and to reflect on the learning journey (VS78, VS79, VS81, VS82). International and intercultural “long-term social relationships” (VS83) can also create changes in perspective and motivation. PHE also needs to incorporate resilience building for learners as they are confronted with dire future scenarios (VS75).

#### 3.2.7. Special role of students in PHE

In the best case, PHE ties in with the real life of students (VS85) and takes up topics that students themselves suggest or are currently concerned with (VS84, VS86). It is also necessary to “take the students seriously, with their questions and concerns, ideas, and suggestions” (VS76). In the development of educational activities, students play an important role because they add a different perspective and have often already dealt with many topics more intensively than (older) lecturers themselves. Students and lecturers can learn from each other (VS88-90). Students can also act as promoters for the implementation of PHE at their universities (VS91).

#### 3.2.8. Need for curricular integration

Extracurricular implementation of PHE is currently clearly predominant (VS93). Many interviewees called for the integration of PHE as a transversal theme into the entire curriculum (VS95, VS101, VS103). Lecturers of different subjects should repeatedly refer to PH within the core medical curriculum by enriching classical medical knowledge with related PH topics (VS96, VS107). PH aspects should not be presented in a disjointed manner, but “build on each other” in a coherent framework throughout the course of medical studies (VS106). In this context, PHE should not be just “an add-on” in the curriculum, but “touches the core of medical studies',” and it corresponds to a “great interest and a great need of the students” (VS99).

Most participants in extracurricular and elective classes or courses are often already sensitized to the topic. Curricular teaching, however, reaches all students, even those who have not yet dealt with topics of PH (VS107).

#### 3.2.9. Innovative and proven didactic methods

Interview participants suggested a wide range of didactic methods in PHE, both innovative and proven. Many suggested combining classroom teaching and blended-learning concepts (VS113, VS114). More discussion and interaction and less “multiple choice” knowledge is required (VS72, VS115, VS117, VS118). Teaching should be interdisciplinary to address the diversity of PH (VS21). Lecture formats would be most appropriate for basic knowledge and best embedded in a broader curriculum with additional seminars in small groups (VS126). Training of communicative competencies, with simulation patients (VS124), problem-oriented learning cases (VS120, VS125, VS128), constructive solution finding with experts (VS129), and excursions with practical relevance (VS64, VS130, VS131), were also mentioned. Examination formats in PHE could include essays on a self-selected topic (VS133, VS136), interviews in group work with various stakeholders (VS123), and a subsequent report that analyzes and reflects on a patient's care environment in group work (VS135). The assessment methods and their evaluation require more time and effort, but promote gaining “a deeper understanding” (VS136, VS135).

#### 3.2.10. Education as a driver of innovation

The overarching concept of PH and its mediation through PHE holds the possibility of driving innovative teaching and research in the field so that each faculty member engages with the connection between his or her own discipline and PH. Educators can develop an awareness of the concept of PH and its complex interrelationships beyond their discipline (VS17, VS142). The goal is “a complete mindset change of the teaching staff” so that PHE is addressed by them in various educational courses (VS137). In this way, the topic could be highlighted also among students, colleagues, and people beyond the medical professions (VS87, VS138-140).

### 3.3. Comparison with three existing frameworks for PHE

When comparing each of the three frameworks for PHE [(19–21), see Table 4] with our ten themes, we found that six of our themes (1.-5.,10.) overlap with two or all three frameworks. We found that two themes (6. and 9.) overlap only with the AMEE Consensus Statement. For two (7. and 8.) of our themes, we did not find a clearly mentioned equivalent in any of the three frameworks. Additionally, we found aspects in the three frameworks, such as “harmony or interconnection with nature” and “indigenous place-based perspectives,” that did not emerge in the interviews.

**Table 4:** Comparison of our ten themes with three international frameworks for planetary health education.

**Themes**	**Cross-cutting principles for planetary health education (20)**	**AMEE consensus statement Planetary health and education for sustainable health care (19)**	**A Framework to guide PHE (21)**
1. Complexity and systems thinking	Systems thinking and transdisciplinary collaborations A planetary health lens, Historical and current global values, *Urgency and scale*	Interconnection of human and earth systems, Complexity and unintended consequences, Dealing with complexity and uncertainty, Systems thinking, *Urgency and scale*	Systems thinking and complexity, The anthropocene and health, *Movement building and systems change*
2. Inter-and transdisciplinarity	Systems thinking and transdisciplinary collaborations	Transdisciplinary collaboration	
3. Ethical dimension	Inequality and inequity, *Historical and current global values*	Respect for human rights and dignity, Equity & social justice, Responsibility for ethical resource use, Challenging inequity & the misuse of power, *Differential impacts of ecological change*	Equity and social justice
4. Responsibility of health professionals		Professional duty to protect health, Eco-ethical leadership, *Good governance, accountability*	*Movement building and systems change, The anthropocene and health*
5. Transformative competencies including practical skills	Organizing and movement building, Communication	Communicating knowledge, Health promotion, Advocacy, *Evidence-based practice*, *Collaborative planning* & *action to mitigate* & *adapt to the ecological crisis (SDG 13.3)*	
6. Space for reflection and resilience building		Dealing with (...) uncertainty, Reflective practice	
7. Special role of students			
8. Need for curricular integration			
9. Innovative and proven didactic methods		Integration of varied forms of knowledge	
10. Education as a driver of innovation	*Organizing and movement building*	Supporting pathways to net zero health care, *Informing policy*	Movement building and systems change
Aspects that have not emerged from our data			
(Acting in) Harmony with nature, Indigenous place-based perspectives	Interconnection with nature
*Aspects from the three frameworks displayed in italics correspond only partly with our themes*			
Dark shading: Overlap of our theme with aspects from 2-3 of the frameworks (themes 1.–5. and 10.)			
Light shading: Overlap of our theme with aspects from 1 of the frameworks (themes 6. and 9.)			
No shading: Theme not explicitly mentioned in the frameworks (themes 7. and 8.)			

## 4. Discussion

To the very best of our knowledge, this study conducted with a large variety of stakeholders at 15 medical schools across Germany is the first study in the German-speaking context that explores high-quality characteristics of PHE through qualitative interviews. Participants reported diverse professional and personal backgrounds, in line with the transdisciplinarity often highlighted in the context of PH (33). Of the ten characteristics we identified, six overlap with two to three existing international frameworks for PHE.

### 4.1. Themes mentioned in two or all three compared frameworks

Complexity and systems thinking refers to the climate and other environmental crises as examples of so-called (super-)wicked problems that pose particular difficulties to public policy, mainly owing to being the emergent outcome of multiple interactions between natural and social systems in what are called complex systems (34, 35). Methods for training students in systems thinking include complex systems mapping (36, 37). Important paradigms that should form part of systems thinking in the context of PHE include the social (and other) determinants of health (38). An approach to understand the complex interrelationships between human health, social and economic factors and the environment can be the doughnut model (39). In medical education it can also be beneficial to explore analogies between the human body as a system of complex systems and the natural complex systems to appreciate the importance of systems thinking for tackling problems and finding solutions.

Inter- and transdisciplinary approaches are highlighted as essential but often lack conceptual clarity at least in relation to the public health workforce (40). Achieving profound changes for PH, such as decarbonizing health care systems, requires collaboration of different professional groups and stakeholders (33).

The ethical dimension includes aspects of equity and social justice in a global perspective (41–43) and questions of climate justice (44). Equally important are questions of ethical resource use in the health sector, with many open questions of implementation that need to be addressed in practice and teaching. Learners can take on an important role of pushing for reflection of current practice in resource waste in their clinical placements.

Regarding the responsibility of health professionals, part of the PHE learning objectives should be the sensitization of medical students to the double-edged role that health (care) systems play for PH including the training of skills for sustainable healthcare (45).

Transformative competencies that would enable learners to effectuate change, were confirmed as a key tenet of high-quality PHE, and also form the basis of high-quality health professional education (46). These can include conventional skills such as communication skills, which can unfold transformative potential if integrated with factual knowledge relevant to PH, for instance regarding the co-benefits of active mobility (meaning health benefits through physical activity as well as less emissions of CO_2_ and air pollutants), a doctor might effectively integrate into a medical consultation. To adopt the role of change agents, learners also need to develop a set of values and attitudes that is in line with the goals of PH. As changes in values are usually not achieved through classic lecture formats but rather through experiential learning and constructive dialogue (47), PHE needs to encompass formats and nudges to trigger this kind of transformative learning in students (48, 49). Developing confidence as well as a sense of self-efficacy are further important competencies that enable students to be agents of change. Faculty can contribute to this by increasing the students' understanding of transformation and their sense that transformative change is possible; enhancing the students' sense of their own agency and ability to make a difference; helping students see and apply PH concepts during rounds or debriefing of clinical encounters; and give support to articulate a role for themselves in processes of transformative change, also by overcoming a perceived gap between the impacts of individual and collective change (50–53). Recognizing and reflecting on the importance of even small contributions to social change in accordance with e.g., the concept of social tipping dynamics (54) can be beneficial to increase learners' self-efficacy and their self-identification as change agents. However, learners should be supported in setting realistic expectations regarding their impact to prevent them from feeling frustrated, discouraged or paralyzed which could result in impaired mental wellbeing.

### 4.2. Themes mentioned in one of three compared frameworks

Our theme space for reflection and resilience building was—among the three frameworks—only reflected in the AMEE consensus statement (19) as learning to face existing and upcoming uncertainties and to implement “reflective practice” as a teaching method. Our category extends this notion by explicitly mentioning the importance for students to be given space to share their emotions and worries in relation to the climate and environmental crises that they might become fully aware of through participating in PHE. Educators have a special responsibility here to consider students' psychological wellbeing when they are confronted both with the scientific evidence on the climate and ecological crises and their health impacts as well as with the implications these might have for their work as medical doctors, but also for their future life (55). Learning critical reflexivity, for example regarding one's personal standpoint, practice, research, and action, including the negative impact of affluent lifestyles (56) can help students to develop and strengthen personal and professional attitudes, and it can strengthen their emotional resilience (57).

### 4.3. Themes not explicitly mentioned in three compared frameworks

The special role of students is not explicitly emphasized in any of the three frameworks. According to our results, high-quality PHE should be student-centered as they are often the first to advocate for changes in their education based on their already existing civic engagement with the climate and other environmental crises (58). Student-led seminar formats can allow students to deepen their knowledge on a specific topic and strengthen their communication skills, thus swapping the traditional roles of student and teacher. A range of student-driven PHE formats have already been successfully realized and can be used as blueprints for further PHE initiatives (59, 60). Students can also play a central role in implementing PHE in the curricula (61).

Conceptualizing PHE as a longitudinal part of the core curriculum is not specifically mentioned in the central graphs of the three frameworks. More recent literature exists on roadmaps for curricular integration, having the potential to reach those who have not previously dealt with planetary health (62). Examples for curricular integration range from simply changing application examples in standard medical lessons (e.g., explicitly describing the effects of heat waves on elderly people or infants) to full lectures on the health impacts of the climate and ecological crises and dedicated teaching for development of practical skills for transformative action on the individual, organizational, and professional-political level (63, 64).

### 4.4. Themes not emergent in our data

The central feature of the PHE framework by Guzmán et al. is “interconnection with nature”. Shaw et al. also refer to interconnectedness with nature as an important element of PHE. Surprisingly, this was not explicitly mentioned by our interview participants when they were asked about quality characteristics of PHE. In our opinion, an understanding of human beings as embedded within natural systems and the unconditional dependency of health and wellbeing on intact and thriving natural systems is essential for promoting and achieving PH.

Shaw et al. also highlight the importance of Indigenous knowledge systems which they claim should be recognized and discussed as part of PHE. This aspect did not emerge from our data either. Here a reason might be that compared to other regions of the world (i.e., America, Australia, Asia, and Africa), Germany has no indigenous populations (in the classical sense of living descendants of pre-invasion inhabitants in a given area that is now dominated by other inhabitants) that would lead a lifestyle close to nature. It is important, however, to also convey this perspective to students in Germany as indigenous populations play an important role in guarding a large proportion of the planet's biodiversity (65, 66) and can bear examples of sustainable lifestyles (67). We believe that the potential of Indigenous knowledge systems for addressing the ecological crises as well as sensitization of learners to the shared drivers of these crises and the marginalization of Indigenous communities and others groups - which can be subsumed under the labels of settler-colonialism, Eurocentrism and extractive economic practices - should be part of PHE. Therefore, more work is needed to sensitize educators in Germany to these issues and to practice dialogue between scholars who were scientifically socialized within different ontological and epistemological cultures.

### 4.5. Strengths and limitations

At the time of publication, the data were almost 1.5 years old. Because PHE is a very dynamic area, some progress may have been made in the development of PHE since we collected the data that is not yet reflected in our paper. We used PHE networks for sampling, followed by snowball sampling, therefore we cannot rule out a selection bias. Moreover, most of the interviewers all had a strong interest in promoting PHE, thereby potentially leading to social desirability bias. We interviewed students as experts who are actively promoting PHE within their medical schools, through which the role of students may be slightly overestimated. On the other hand the inclusion of diverse stakeholders, from 15 universities from all over Germany and specifically the inclusion of students with an active role in PHE—whose contribution is essential—allowed us to gain a holistic understanding of current priorities in this dynamic field.

## 5. Conclusions and further implications

The ten characteristics of PHE we developed from interviews with a diverse group of stakeholders at medical schools throughout Germany can be helpful to all who are currently in the process of implementing and enhancing PHE nationally and internationally. While most of our findings were in line with existing frameworks, we also identified new themes. Focus should be laid on the special role of students, space for reflection and resilience building and transformative competencies. Further studies should focus on other health professions to meet the aspiration of inter- and transdisciplinarity in the design and underlying principles of PHE.

## Data availability statement

The raw data supporting the conclusions of this article will be made available by the authors, without undue reservation.

## Ethics statement

The studies involving human participants were reviewed and approved by Ethics Committee of the University Hospital of Würzburg file number 20210312-01. The patients/participants provided their written informed consent to participate in this study.

## Author contributions

E-MS-S conceptualized the study. E-MS-S and JS planned the study and performed the data collection and the thematic qualitative text analysis. E-MS-S, JS, and KW performed the comparison of the themes to existing frameworks. AS supervised the whole project (doctoral thesis of JS). All authors contributed to the manuscript development and approved the final version to be published.
