# Supplementary material for: Ten characteristics of high-quality planetary health education—Results from a qualitative study with educators, students as educators and study deans at medical schools in Germany
Source: Front Public Health. 2023 Apr 25;11:1143751. doi: 10.3389/fpubh.2023.1143751 (PMC10166869; doi:10.3389/fpubh.2023.1143751)
Supplement: Supplementary file 1 [file Data_Sheet_1.pdf]

Supplementary Material

Ten characteristics of high-quality planetary health education – Results from a qualitative study with educators, students as educators and study deans at medical schools in Germany

Johanna Simon, Sandra Parisi, Katharina Wabnitz, Anne Simmenroth, Eva-Maria Schwienhorst-Stich*

*** Correspondence:** Corresponding Author: schwienhor_e@ukw.de

S1: Study design PlanetMedEd
S2: English translation of the semi-structured interview guide
S3: COREQ – Consolidated criteria for reporting qualitative research checklist
S4: Coding tree – Codes and Subcodes
S5: English translation of verbatims
S6: Original German verbatims

S1: Study design PlanetMedEd

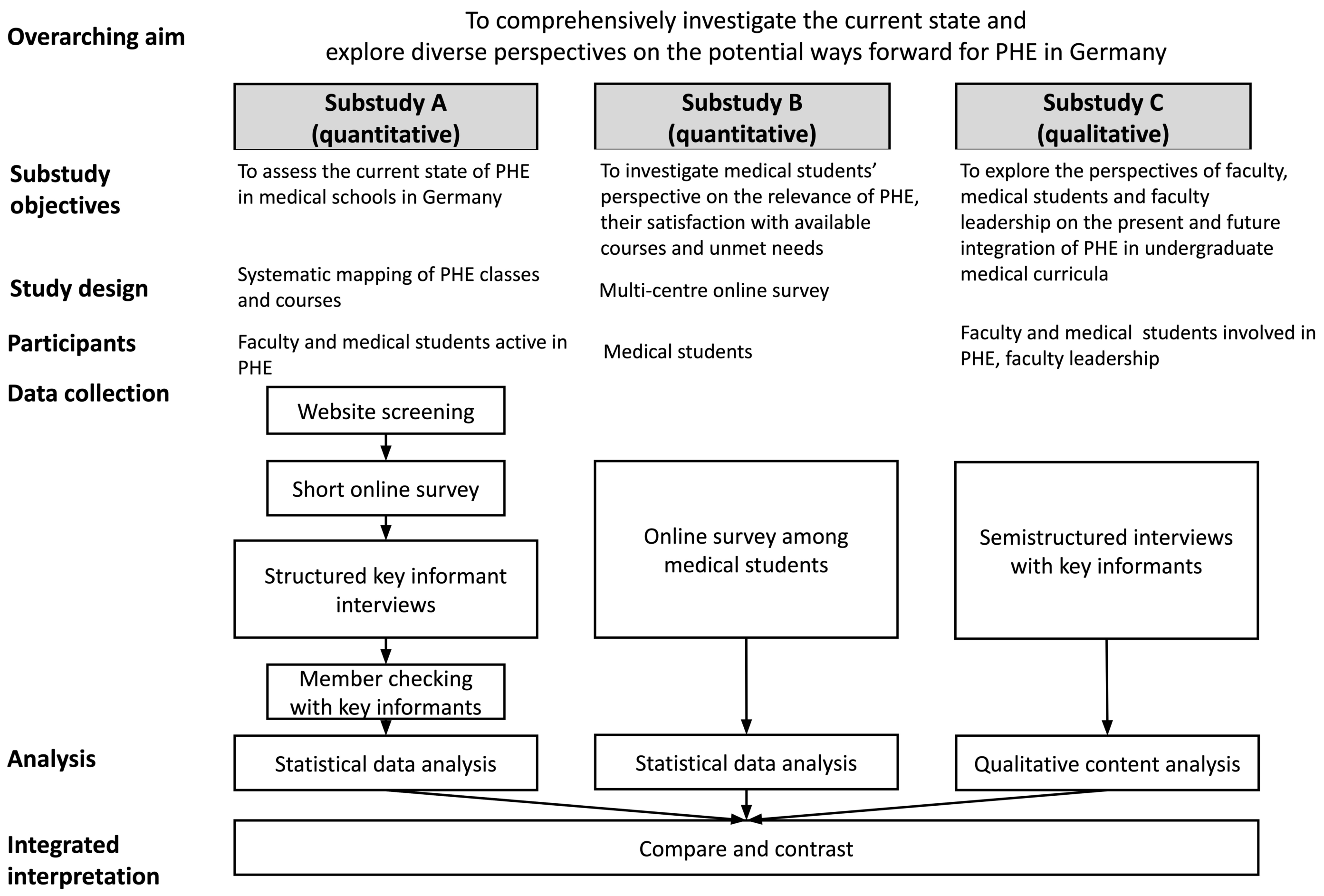

S2: English translation of the semi-structured interview guide

Interview guide

Date	
Duration of the interview	
Name of interviewer	
Interview no.	

1. **Introduction**	
Welcome	Welcome to the interview. We are pleased that you are participating in our study.
Explain the aims of the study	This study is part of a mixed-methods study, i.e. it consists of a quantitative and a qualitative sub-study. In the quantitative part, we want to record which teaching offers on PH already exist at medical faculties in Germany.<br>In this qualitative part, however, the focus is on your personal experiences.
Clarify time frame	The interview will take about 20-30 minutes. But more time is always possible if you want to tell more.
Voluntariness, confidetiality	As already described in the consent form, your participation is voluntary. You can withdraw from the interview at any time. We will record the audio track of the interview. The data will then be transcribed anonymously and the audio file subsequently deleted.
Declaration of consent	We already recieved the signed consent form.

Procedure of the interview	The interview will go as follows: we will start by talking about your experience in PH. Then at the end we would like to ask you a few questions about yourself.
Do you have any questions up to this point? If not, **let's start recording now.**	

1. Questions about the experience

Topic	Key question	If necessary, further questions
**Experience of teaching with PH in general**	First, I would like to ask you to tell us what experiences you have had with PH teaching. Please tell us everything that comes to your mind.	
**Definition teaching in PH**	What does PH mean to you?<br>What constitutes good teaching in PH for you?	Where do you see a focus, if at all?<br>Do you see the focus exclusively on climate change (if mentioned)?<br>What competences should PH teaching be about?<br>(if mentioned: what other competences should participants learn?)<br>To what extent - if at all - do you see teaching PH as different from classical medical teaching?<br>How do you see the relationship between curricular and extracurricular teaching?<br>Where do you see curricular anchoring points in medical studies?<br>Enquiry if aspects of global health are mentioned: To what extent do you see a difference between Global Health and PH and, derived from this, how does teaching in Global and Planetary Health differ?<br>If applicable, ask in the context of the answer about involvement in teaching.
Paraphrase: Let me summarize: PH for you means ...<br>*If talking about Global Health: The following questions refer only to the teaching on PH.*		
**Influencing factors**	From your experience, what are (what would be) for you factors that influence the establishment of teaching programmes on PH?	Can you think of any other factors, e.g. at another level?<br><br>Factors influencing the establishment of new teaching and assessment formats? (e.g. transformative action, if mentioned)<br>Comparison with the usual procedure of curricular adaptation/development of curricular teaching?<br>What role - if any - does the publication of the NKLM 2.0 play for you in this context?<br>caveat: differentiate here between "Global Health only" and Planetary Health

**„Lessons learned“**	Based on your experiences, what do you think should happen to improve and further anchor the teaching of PH?<br>We are interested in everything that comes to your mind, both aspects in your direct environment and on a higher level.	Is there anything you would have done differently in retrospect?<br>Is there anything that went particularly well?<br>*Caveat: if interviewee here is referring to Global Health teaching, ask to transfer experience gained in the context of Global Health teaching to PH teaching.*
**Advice**	What advice would you give students who want to have more teaching on PH in their curriculum?<br>What advice would you give lecturers?<br>What advice would you give decision makers in the faculties?	
**Further interview partners**	From your point of view, can you name other interview partners we should write to? With a complementary view and also at another location?	
**Participation in quantitative sub-study**	Has the person conducting the structured interviews already contacted you about participating in the structured interviews?	

1. **Questions about the person**
Now we would like to ask you a few more questions about yourself:<br><br>o Student:<br>How far along are you in your studies? How long have you been teaching?<br>o Lecturer:<br>How long have you been working professionally? / How many years of professional experience do you have? How many years have you been a lecturer?o<br>Teaching decision-makers:<br>How long have you been in your current position?<br><br>Can you very briefly tell us about your academic training and professional focus?Are there any other personal or professional experiences, backgrounds or qualifications in the context of teaching or PH that you would like to mention?<br><br>Lastly, we would like to record what gender you identify with and what age decade you are?

3. Record important impressions:

4. Reflection

- Went well? Own feeling?
- Time (how long did the interview take?)
- Did I influence the interviewee(s)?
- Were there any problems in understanding?

Topics/Participants

- Which (over)topics were mentioned, were there any new topics?
- Is there overlap with other interviews, saturation?
- Are there any new topics that should be included in the interview guide?
- Are there new participants (groups) who should be interviewed?

S3: COREQ – Consolidated criteria for reporting qualitative research checklist

No	Item	Guide questions/description	Additional Information
**Domain 1: Research team and reflexivity**			
Personal Characteristics			
1.	Interviewer/facilitator	Which author/s conducted the interview or focus group?	E-MS-S, JS
2.	Credentials	What were the researcher's credentials? *E.g. PhD, MD*	E-MS-S: MD, MScIH<br>JS: doctoral thesis student
3.	Occupation	What was their occupation at the time of the study?	E-MS-S: Department of General Practice/Family Medicine, University Hospital Würzburg;<br>JS: doctoral thesis student
4.	Gender	Was the researcher male or female?	Both female
5.	Experience and training	What experience or training did the researcher have?	E-MS-S: Experience in PHE and implementation research

No	Item	Guide questions/description	Additional Information
Relationship with participants			
6.	Relationship established	Was a relationship established prior to study commencement?	Contact via Email, informed consent, partly collaboration in working groups
7.	Participant knowledge of the interviewer	What did the participants know about the researcher? e.g. *personal goals, reasons for doing the research*	Study Objectives; Study part of doctoral thesis; Engagement in PHE
8.	Interviewer characteristics	What characteristics were reported about the interviewer/facilitator? e.g. *Bias, assumptions, reasons and interests in the research topic*	Engagement in PHE, doctoral thesis
**Domain 2: study design**			
Theoretical framework			
9.	Methodological orientation and Theory	What methodological orientation was stated to underpin the study? *e.g. grounded theory, discourse analysis, ethnography, phenomenology, content analysis*	Thematic qualitative text analysis with a content-structuring approach according to Kuckartz

No	Item	Guide questions/description	Additional Information
Participant selection			
10.	Sampling	How were participants selected? *e.g. purposive, convenience, consecutive, snowball*	Purposive, snowball
11.	Method of approach	How were participants approached? e.*g. face-to-face, telephone, mail, email*	Email
12.	Sample size	How many participants were in the study?	20 participants
13.	Non-participation	How many people refused to participate or dropped out? Reasons?	10 (no answer to email, not the right contact person)
Setting			
14.	Setting of data collection	Where was the data collected? e.*g. home, clinic, workplace*	Home/workplace via Zoom®
15.	Presence of non-participants	Was anyone else present besides the participants and researchers?	In one interview another member of the research team
16.	Description of sample	What are the important characteristics of the sample? *e.g. demographic data, date*	See Table 1
Data collection			

No	Item	Guide questions/description	Additional Information
17.	Interview guide	Were questions, prompts, guides provided by the authors? Was it pilot tested?	Yes, and pilot tested
18.	Repeat interviews	Were repeat interviews carried out? If yes, how many?	No
19.	Audio/visual recording	Did the research use audio or visual recording to collect the data?	Audio recording via voice recorder
20.	Field notes	Were field notes made during and/or after the interview or focus group?	Yes
21.	Duration	What was the duration of the interviews or focus group?	13-43 minutes
22.	Data saturation	Was data saturation discussed?	Yes
23.	Transcripts returned	Were transcripts returned to participants for comment and/or correction?	No
**Domain 3: analysis and findings**z			
Data analysis			
24.	Number of data coders	How many data coders coded the data?	2 (E-MS-S, JS)
25.	Description of the coding tree	Did authors provide a description of the coding tree?	See Supplementary Material S3

No	Item	Guide questions/description	Additional Information
26.	Derivation of themes	Were themes identified in advance or derived from the data?	Both (Thematic qualitative text analysis with a content-structuring approach according to Kuckartz)
27.	Software	What software, if applicable, was used to manage the data?	MAX-QDA 2020
28.	Participant checking	Did participants provide feedback on the findings?	No
Reporting			
29.	Quotations presented	Were participant quotations presented to illustrate the themes / findings? Was each quotation identified? e.g. *participant number*	Yes, see additional verbatim
30.	Data and findings consistent	Was there consistency between the data presented and the findings?	See Results
31.	Clarity of major themes	Were major themes clearly presented in the findings?	See Results
32.	Clarity of minor themes	Is there a description of diverse cases or discussion of minor themes?	See Discussion

Tong A, Sainsbury P, Craig J. Consolidated criteria for reporting qualitative research (COREQ): a 32-item checklist for interviews and focus groups. *International Journal for Quality in Health Care* (2007) 19:349–357. doi: 10.1093/intqhc/mzm042

S4: Coding tree – Codes and Subcodes

Code	Subcode	description
**Complexity and systems thinking**	Content and knowledge base	Challenge of the breadth and depth of topics with the aim of gaining basic knowledge and historical understanding
	Systems thinking	Learning a way of looking at the multiple interrelationships of the individual (earth) systems
**Inter- and transdisciplinarity**		Inter- and transdisciplinary collaboration is essential for quality PHE
**Ethical dimension**	Ethical assessment	Discuss and evaluate ethical issues
	Social justice perspective	Knowledge of global differences regarding social justice and its ethical reflection
	Travel/consumption behavior	Reflection and discussion of own travel and consumption behavior
	Think global, act local	Awareness that local behavior also has a global impact
**Responsibility of health professionals**	Awareness of responsibility as a physician	Awareness of one's responsibilities as part of the health care system (at the micro, meso, and macro levels)
	Professionalism, Attitude	Basis for the professional appearance of physicians in society
**Transformative competencies including practical focus**	Motivation and confidence in one's own options for action	Support students in their motivation, find solution-oriented ways and apply them in a practical way in order to strengthen confidence in their own options for action
	Scientific work	Learn critical, reflective, evidence-based scientific work
	Communication skills	Learning of communicative competences, both towards patients and fellow citizens
	Space for reflection	Provide space to reflect on what has been learned, to put it into context

**Space for reflection and resilience building**	Social relationships	Establish social relations in countries and regions to enable exchange
**Special role of students**	Adapt teaching to students	Teaching must be linked to the topics that students are concerned with in their everyday lives
	Students as lecturers and teaching developers	Students play an important role not only as learners, but are often the driving force for more teaching in PHE and also lecturers themselves in electives
**Need for curricular integration**	(Extra)-Curricular ratio	What is and should be the relationship between curricular and extracurricular teaching?
	Curricular anchoring points	Where and how can PHE content be curricularly integrated?
	Driving discipline	Which department at the medical school might best be responsible for establishing PHE and why
**Innovative and proven didactic methods**	General quality of teaching	General quality in teaching, not necessarily specifically related to PH
	Comparison PHE and class. med. teaching	In what ways is teaching in PG different from traditional medical teaching?
	Teaching formats/methods	Which teaching formats and methods are suitable for PHE?
	Exam formats	Which examination formats are suitable for PHE and are possibly already in use?
	Comparison Planetary Health Education and Global Health Education	To what extent do PH and GH (Global Health) differ and, derived from this, to what extent do PHE and GHE differ?
**Education as a driver of innovation**	Overall concept	Concept that keeps popping up in various places, closes a frame around all medical teaching (and beyond)
	Radiance/taking others along	Joint process of establishing teaching reaches people from different disciplines, even if they have not previously known about and dealt with the subject at all
	Mindset change faculty/create awareness	Awareness among faculty can create a general mindset shift, which in itself can get more projects rolling

S5: English translation of verbatims

1. Complexity and systems thinking	
VS1	That's why I think it's still a big challenge to somehow set up a good program where you have a common thread and still somehow go into depth, because up to now I've had the feeling that it tends to stay on the surface. (P1)
VS2	At some point, of course, this becomes so broad and so arbitrary that it is very difficult to draw any boundaries at all. That is certainly also a danger in all of this. (P8)
VS3	I think that's the attraction, but also the difficulty, that it's so wide. (P10)
VS4	to put the current situation into historical perspective. [...] that we are really in a phase, as I indicated at the beginning, if we look back at how the development of modern medicine and modern society began 150, 200 years ago, a development has simply established itself in the global North in the colonial structure, which is really pathogenic (P3)
VS5	Topics such as communication, such as social aspects, such as environmental relations and medical geography. (P3)
VS6	And we structured it in such a way that at the beginning we first developed a common understanding of PH and then in the course of the event we focused on different aspects, for example, what are the connections between climate change and health, what are the specific diseases, for example allergies, and how are they connected to climate change or heat waves. (P2)
VS7	But above all, it is again about... yes, of course, about factual knowledge. (P3)
VS8	Of course, you first have to learn a little bit about what PH is, why it's important, that would have to be told to you by someone who knows about it, as a kind of lecture. (P6)
VS9	For me, it's first of all the basic understanding of [...] climate change and from that understanding the impact on human health. (P16)
VS10	But at the end of the day, for me in Planetary Health (PH) it's always [...] about the impact on patients and of course the preventive aspect, what can we do in the health care system to mitigate climate change. (P16)
VS11	I think it's very important that you don't cut it down to environment and health, but that you include a lot of things, how everything is connected. (P5)
VS12	I always find it difficult in the lectures we have at university when you only look at one aspect or one thing. But it's a huge system, and in order to understand it, I think everything has to be illuminated in some way, and partly also non-medical things. (P5)
VS13	that you don't shorten it and that you somehow accept this whole, [...] huge system, which you can't understand at all because it is so big (P5).

VS14	if you want to understand [...] the system of climate change, [...] then you don't have to work through all the examples and then know all the examples, but it is enough to work through a few and then you simply have an understanding of how such structures work and how you can rethink such things. (P5)
VS15	It goes far beyond the classical medical curriculum. Also the concept: the planet is sick, let's see what the symptoms are. (P10)
VS16	to open the eyes of students and future physicians to the fact that health does not end at the hospital door or at the practice door, so to speak, but that everything that happens in our lives and in our everyday life influences health and that it is then also influenced globally. (P11)
VS17	that one simply develops an understanding that the part we call medicine [...] is often a very small part of what actually defines the health of all of us, and above all will also define health in the future. (P11)
VS18	Of course, you have to communicate basic knowledge about global climate and environmental changes, [...] and then you also have to make sure that the students come up with these cross-connections on their own. (P12)
VS19	Thinking outside of silos. Looking at the whole [...] seeing people as a whole in the environment. I think that's the competence that's very much in the focus there. (P14)
2. Inter- and transdisciplinarity	
VS20	but I think, especially in the case of planetary health, it is enormously wide-ranging and [...] we also had speakers from the most diverse areas. (P1)
VS21	it differs above all in the interdisciplinarity, because [...] in classical teaching, of course, there are also subjects that are already very interdisciplinary, where we also include a wide variety of fields, but I think, especially in planetary health, it is just enormously wide-ranging. (P1)
VS22	you can only try to take the various diverse facets into account and that's why it's interdisciplinary, and you just have to work with colleagues who have other perspectives and ideas and with people who have other perspectives and ideas. (P3)
VS23	For example, I think it would be cool if we did something with agriculture and also with [...] farmers together or something. [...] With those who feel threatened in their existence. You have to do something together with them, you actually have the same goals. (P3)
VS24	if the lecturers came from a wide range of disciplines and perhaps also the learners did not belong to just one discipline, because it is an interdisciplinary subject. And if you really want to approach each other and make better contacts, then it would only be logical to learn together in the different disciplines with the different disciplines. (P4)
VS25	To understand it, you have to look into other scientific areas, into social areas and cultural things. (P5)
VS26	I thought it was great to sit down with geographers, geologists, student teachers, psychologists, and talk about teaching didactics. And I

	found it very enriching. And then I already had this transdisciplinary idea, which plays an enormous role in PH. (P8)
VS27	but I rather have the impression that it involves a common understanding of what we do in medicine. Across all professions. (P10)
VS28	This is, of course, a big topic that concerns interprofessionality, interdisciplinarity. (P10)
VS29	The main difference from my point of view is now the inclusion of other groups and members of society that have not been classically considered by us so far. (P10)
VS30	Good teaching in PG is highly inter- and transdisciplinary. Because you cannot give a lecture as a dermatologist in the field of PG, but you have to work together with interdisciplinary colleagues or with colleagues from other disciplines, which means that the PG lecture should be an interdisciplinary lecture. But in the end that would be [...] the prime example of an interdisciplinary lecture, where you should also have geographers reading, where you should also have space researchers reading and so on and so forth. (P14)
VS31	and when we have this interdisciplinary teaching, which always thinks about the end, that is, about the prevention of people, so that people stay healthy in the end. Then this kind of teaching, which we choose for PH, [...] should radiate into other disciplines (P14).
3. Ethical dimension	
VS32	and also be able to evaluate the whole thing ethically. (P9)
VS33	the essential difference is, of course, [...] that in PG and climate change we ultimately discuss [...] issues of existential importance and, of course, the ethical implications are definitely more far-reaching and more difficult to weigh than in a classical natural science subject. (P9)
VS34	Instead, we are endeavoring, especially in the German healthcare system, to do things that already consume resources. So the question is whether chemotherapy, if I give it, might not already have a very bad CO2 balance, can I make up for that at all. But then again, these are also ethical questions. (P16)
VS35	But while working on the content, doing so to address these fundamental principles. Like social justice, for example. [...] Or also the big topic of gender and sex, or also racism, sexism[...] And also at the same time a [...] critique of capitalism is also quite essential for me. (P17)
VS36	that you think carefully, according to which basic principles can we somehow arrange our society. (P17)
VS37	How, for example, social inequality and the other things [...] are involved and present in all the different environmental-health interactions. And, that it's involved, that's actually the essential thing for me. (P17)
VS38	Planetary health is also related to the social reality in the world, that one does not only see it from a scientific point of view, et cetera, and from the perspective of the Global North, but it is also related to the social issues, both social inequality here with us related to

	climate change and also [global] climate change consequences. (VS38, P3)
VS39	You can't do Global Health anymore without Planetary Thinking. And it makes just as much sense to talk about, I think, Planetary Health if you don't take into account issues of Global Justice and Health Systems and all the classic Global Health issues. (P3)
VS40	I think this is a topic of PH, which is not yet considered so strongly among us, which is why I find this global and also the social and questions of social justice, global justice perspective very important. (P3)
VS41	Yes, maybe also again an understanding of inequality, how for example climate change affects different regions differently. (P4)
VS42	perhaps also a reflection [...], to how far we have not yet really decolonized global health and so on. (P4)
VS43	with this kind of sustainability commission, where we really work out a business travel and travel concept for the university, where we also include the students in order to simply consider when it is actually still justified to take a flight. (P3)
VS44	And avoiding air travel completely and otherwise continuing to live as before doesn't help either, that's a bit of a pseudo attitude: yes, I won't fly anymore, right? And that is then also partly very superficial, yes, one must also think more comprehensively, more multidimensionally and really reflect on one's own actions, but not remain on the surfaces of "I won't do that anymore" or "[...]no one is allowed to eat meat anymore". (P3)
VS45	to think about health in such a way that our actions here also have an impact worldwide, so this “think local, act global”. (P2)
VS46	think globally and act locally, truly feel responsibility locally as well, and do local initiatives. (P3)
4. Responsibility of health professionals	
VS47	critical thinking and reflection, where you reflect your own point of view, your own position, that is your own positioning in our society, in the global society, referring to all the issues that are important for PH. (P3)
VS48	we also have a contribution to it and are also partly to blame or partly responsible for it or are also privileged in the position to change anything about it. (P5)
VS49	It (PHE) must clarify in any case for the students the significance they can have as physicians later in their professional life for this issue, and students must be aware that it makes an immense difference whether they perceive this responsibility or not. (P7)
VS50	One of the main competences would really be that students later on are able to critically reflect on their medical actions again and again... that is, they are able to critically reflect on the effects of their medical actions with regard to mechanisms that either serve or stand in the way of the health of our planet. (P8)
VS51	but that I perhaps also reflect, for example, on my entire practice management, yes, where do I get my electricity from, yes, do I necessarily always have to drive to home visits or is an e-bike perhaps also sufficient, so that I also have a holistic view of my own

	actions. (P8)
VS52	Doctors often tend to always give smart advice to others, but of course we can start with ourselves, what we can do about it ourselves. (P8)
VS53	That they (learners) can deduce and discuss what consequences certain actions have, or even non-actions have. (P9)
VS54	how strongly we are all also role models in our behavior as citizens. (P10)
VS55	I personally find it totally important to somehow have an awareness of the social responsibility [...] that one bears in this profession (health sector), this [...] sense of duty and responsibility. (P12)
VS56	What does that mean for my professional goal that I'm striving for, but what does it also mean for my personal life? (P13)
VS57	and on the other hand also question our own actions in the medical sector and learn what effects our actions and [...] the prescription of different drugs, things like that, have. (P15)
VS58	I'm not even sure whether this can be captured primarily through a concept of competence, well, I think that my understanding of role models and competences here is the best way to connect some kind of... professionalism with it. (P10)
VS59	In that sense, I think it's more of an attitude question and a framing question, almost a culture question. (P10)
VS60	everyone must have heard about it and develop an understanding of it. Attitude for all and immersion for those interested [...] medical students as critical citizens and physicians as responsible critical citizens in a broader sense. (P10)
5. Transformative competencies including practical skills	
VS61	And can also point out the positive things about it, that is, what happens [...] if we change this and that, how does that have a positive effect. (P5)
VS62	And that it can also be super motivating... compared to other subjects (P5).
VS63	Good teaching is more than simply learning facts by heart. Good teaching also includes, in particular, conveyance of the contexts and transformation of them into knowledge for action, so that students can actually apply this knowledge in their own actions, that they can also reflect on the knowledge, that they can deduce and discuss what consequences certain actions or non-actions have. (P9)
VS64	but also to say that we offer cooking courses for medical students, together with another profession, make this practically manageable and also consider how someone can do this at all under certain social conditions. And maybe we go shopping together with people from the school, or... So to really bring such things into an action, into an action that is also beyond the classic fields that we play in, so far, that would be interesting to me. So, that we think more, well, outside the classical boundaries. Maybe a visit to a farm, how does food production work, what factors play a role. So this is a very fundamental problem that doctors have only a very superficial overview of so

	far, I think. (P10)
VS65	a critical perception, so also, how can I critically question things that happen,[...] how can I translate things that happen in the environment or globally, for me, for my daily experience, for my daily actions? [...] and then also a bit of solution-oriented thinking, [...] how can you work forward, what can I also contribute? (P11)
VS66	It is first of all the basis, so to speak, for the understanding and then it can go on with approaches to solutions, creative... yes, transformative ideas. (P12)
VS67	the coordination between what you can work on in guided self-study and then implement, apply, discuss, deepen in contact study appropriately prepared. And that is independent of which topic, or whether it is only pure knowledge transfer, or perhaps application knowledge, inclusive practical knowledge, I think you can think this blended learning concept, this distinction between guided self-study and contact study in all areas. (P16)
VS68	that the students think a bit creatively and also simply freely, how do we want to have that, without the, yes, I'll say now, shackles of the way it is now. In order to draw the strength from this: yes, we can also somehow change the world the way we would like to. (P17)
VS69	That is scientific work and I would expect that you really do it from a scientific perspective, which simply means that you look at the facts carefully and critically, even if they don't suit you, and bring different perspectives into connection with each other and then try to find a coherent, plausible intermediate solution. (P3)
VS70	A physician, also in the role of a scientist, must be able to evaluate scientific findings, to assess them correctly, also with regard to the sources, for example, to find them in the first place, to classify them, to reflect on them, to communicate them and to be able to deduce one's own options for action from this knowledge. (P9)
VS71	that you have the possibility to deal with a topic more intensively, that you have the possibility to inform yourself, that means that you learn, how can I use sources, how do I get to good sources on such a topic? (P11)
VS72	but also the political, social, communicative aspects. And you can't just do that in a multiple choice way, so a few facts, of course, there are simply facts that you can also learn, you just have to have them. But in the meantime we also know, and this is also shown by the pandemic, that simply because we have facts and accept them, we still don't know what to do and how to communicate it, and we don't achieve any behavioral and political changes. (P3)
VS73	that perhaps one actually thinks again about this extracurricular idea outside of these two zones of study, that is, that one really says that we have the task of being more visible to our students in society. At schools, in old people's homes and nursing homes [...] Maybe also to actually talk to people in the street and to discuss, [...] we don't practice that at all. (P10)
6. Space for reflection and resilience building	
VS74	For me, good teaching means that the students are enthusiastic, that they come up with their own ideas, that they are allowed to discover

	and develop their own thoughts, that they are allowed to look for solutions themselves, that they are also encouraged in the process, and that they are not given a path to follow, but that they have room to develop things. (P2)
VS75	that it's not something that should be so school-like, but that it really aims to, yes, make their own thoughts and also offers them a space to reflect on their own behavior, and are also accompanied in the process. That's what I think makes the teaching there, [...] so that they are also accompanied in the findings that are gained on the way and that are sometimes very sobering, frustrating or also... yes, can also move you very much. (P2)
VS76	And above all, I think you have to take the students seriously, with their questions and concerns, ideas and suggestions. (P3)
VS77	problem-oriented learning, thinking for oneself, critical thinking, where not only facts are asked that one has learned by heart in the exams, but critical thinking is simply required. (P6)
VS78	that they can also reflect on the knowledge (P9)
VS79	how can I critically question things that happen, [...] how can I translate them, so to speak, so how can I translate things that happen in the environment or that happen globally, for me, for my daily experience, for my daily actions? (P11)
VS80	systemic thinking, critical thinking, one must of course somehow try to promote. To simply question things and [...] to somehow broaden the horizon. (P12)
VS81	first and foremost, for me, it's about how can students and future doctors [...] go into the interaction and treatment of, with patients, with open, watchful eyes and a reflective understanding. (P16)
VS82	to question the seemingly self-evident[...] And simply... not to take it for granted, but [...] simply to question everything, no matter what it is, and also to check whether it corresponds to how we actually want to shape the world and live. (P17)
VS83	That we see our work, also on health, also teaching and something like that, in pure form also as social relationships, that we have to do with people, not only with objects, neither with the students nor the people with whom we work somewhere abroad, or whatever, they are all people [...] And long-term social relationships are simply also what carries us and motivates us. (P3)
7. Special role of students	
VS84	To connect with the students where they are at the moment. In other words, to link to topics that they might also throw into the classroom and then to make the connection to planetary health. (P8)
VS85	I think that it makes no sense to treat the subject dogmatically, but that it is primarily the case that I think you also have to get the students involved in the subject. And that is the beginning of good teaching on the subject, that it connects well founded to the life world of the students. (P13)

VS86	Good teaching in this area, yes, should on the one hand dock onto the students' lifeworld, should bring them into the subject, and should then also be linked back again and again in a practice-oriented way. (P13)
VS87	Beyond that, however, I think - and that's why I'm also strongly in favor of it being as present as possible as a cross-cutting topic in all events - that we also have young people in our studies who we accompany a bit on their life's journey, and with whom we simply have to set the topic. (P13)
VS76	And above all, I think you have to take the students seriously, with their questions and concerns, ideas and suggestions. (P3)
VS88	[...] we could always use more space in which exchange takes place, because many (students) have prior knowledge in different areas and we can also learn from each other, so not only the lecturers can teach the students something, but also the other way around. (P7)
VS89	that you might even work it out together with the students, because some are of course already further along, many at our age, and the younger ones are probably already much more concerned with it than the older ones or the lecturers in part, and getting them on board is certainly also good. Because many also have a different perspective (P12).
VS90	very interactive formats, where the students are given a topic and then simply inform themselves comprehensively and then present that, because they then usually become greater experts than the lecturers. [...] this seminar character, where the student becomes the lecturer. (P14)
VS91	This is certainly also a generational issue and possibly also a bottom-up strategy that comes from students and some interested people. (P16)
8. Need for curricular implementation	
VS92	that there are simply not yet any concepts that have been worked out, that are anchored in the curriculum and have already been tested for many years, where one could somehow copy something, but rather everyone is still doing something for themselves that they see as important. (P1)
VS93	the curricular offerings are still very sparse, very understaffed, and the extracurricular has become, I think, huge lately (P2)
VS94	I think that we (...) have to do a lot more there, yes, because I think a lot of people are still not really aware [...] of what it will actually be like here in 20, 30 years. I think that in many areas of society we are not prepared for the fact that we will have significantly higher temperatures, or that we will have something like heavy rainfall events. (P9)
VS95	and therefore this topic should take a broader [...] place. [...] either as a separate event format or as a longitudinal curriculum. (P9)
VS96	structurally, curricular teaching would be extremely appropriate. And, quite often, to establish the link between the things that we learn in conventional medicine, I would say, and what that has to do with PH. (P15)
VS97	They are nowhere near implementing this across the board in the curriculum, but it is important that we build up a nucleus here and then

	also make the project or the content transferable and perhaps roll it out systematically in the future. (P16)
VS98	I think it is very important that it is also offered in the curriculum. [...] so that there is simply the capacity in such a loaded study program, in such a full study program, and that above all also reaches all people [...] who do not yet have so much to do with it. (P17)
VS99	And that is also an important point for me, that PH, just like Global Health, is not something that is only added to medical studies as something completely new that would otherwise be separate from medical studies in itself and is only an add-on, an add-on, an add-on, but it is clearly something that touches the core of medical studies and maps many competencies that are important for medical training, and modern medical training extremely well and also corresponds to a great interest and a great need from the side of the students. (P3)
VS100	that this is simply also represented much more at our university. (P5)
VS101	That it be included in the curriculum as a cross-cutting area. (P5)
VS102	that PH is implemented where it [...] has an effect as a subject, for example in medicine, where it is a lot about: how do diseases develop, how can I work preventively... searching for causes. And there it is very much about lifestyle, where PH is actually a very central topic, but unfortunately it is not yet represented in teaching, because at the moment we are unfortunately only taught secondary and tertiary medicine, a little bit, but very little prevention work. (P6)
VS103	for me PG would be similar to Global Health, if you will, like in English a cross-cutting issue, which should drag practically through the complete teaching plan, through the complete curriculum. (P8)
VS104	I am of the opinion that there are undoubtedly topics that must be included in the mandatory curriculum for everyone with regard to this broader PG. I can imagine that this is also well conceivable as an elective area, especially in the perhaps international context in various perhaps but also regional contexts. (P10)
VS105	If you try to integrate this into as many departments as possible, yes, as is also being attempted in the NKLM, to always draw these cross-connections, that it is simply made more visible and people somehow have to make the connection again and again, so to speak, that everything that actually happens on a small scale is connected to something larger. (P12)
VS106	that it is a matter of first of all making it more visible, the entire subject in a longitudinal orientation, that, so to speak, not only individual topics pop up in medical studies, here is a lecture on it, there is something on prevention, but that they build on each other. (P12)
VS107	that it is actually urgently necessary that all students are confronted with the topic and that it is included in the, yes, standard curricula, in the mandatory courses. And that it is perhaps more goal-oriented, in addition to an introductory course on the topic, to dock it again and again in all clinical and preclinical courses. (P13)
VS108	Ideally, I would imagine a longitudinal teaching concept or a modular concept. [...] of course the timetable is overcrowded at all points.

	And new elements also mean that we have to put a time burden on students, or even cut other proven topics. We are not at that point yet, because this topic is newly established and we are currently making it available to students as an elective add-on. (P16)
VS109	i can't say it fits in one semester and not in the other. Because the topics are so superordinate. It might be good to evoke some basic understanding of the issues already in preclinical and then in the clinical section discuss more the direct implications for diseases, pharmacotherapy et cetera. (P16)
VS110	Because environmental medicine is also exactly that (like PG), we care about how environment makes sick or environment keeps healthy and how we can do prevention in the end. So that means we have to bring environmental medicine more into the curriculum, it's way too far out there and then it should be curricular teaching. But environmental medicine can take over the coordinating role here. (P14)
9. Innovative and proven didactic methods	
VS111	this is no different from other teaching, of course, what you teach has to be technically correct and right. (P3)
VS63	Good teaching is more than simply learning facts by heart. Good teaching also includes, in particular, conveying the contexts and transforming them into knowledge for action, so that students can actually apply this knowledge in their own actions, that they can also reflect on the knowledge, that they can deduce and discuss what consequences certain actions or non-actions have. (P9)
VS112	I think that classical teaching has had its day anyway. We have to revolutionize teaching everywhere anyway. (P14)
VS113	Good teaching is not subject-specific. Good teaching is [...] simply a didactically elaborate and fine-tuned system that also includes blended learning. So for me, pure face-to-face teaching has been outdated since Corona at the latest, and I think it's good to distinguish between what serves to gain knowledge and what serves to transfer knowledge. (P16)
VS114	Because PHE is now implemented de novo, I think it has to have even more sophisticated didactic concepts. I don't think a classical approach would do justice to the topic. Instead, it must be highly innovative, just like the topic itself. (P16)
VS21	it differs above all in the interdisciplinarity, because [...] in classical teaching, of course, there are also subjects that are already very interdisciplinary, where we also include a wide variety of fields, but I think, especially in planetary health, it is just enormously wide-ranging. (P1)
VS72	but also the political, social, communicative aspects. And you can't just do that in a multiple choice way, so a few facts, of course, there are simply facts that you can also learn, you just have to have them. But in the meantime we also know, and this is also shown by the pandemic, that simply because we have facts and accept them, we still don't know what to do and how to communicate it, and we don't achieve any behavioral and political changes. (P3)
VS115	I think that you can also acquire completely different competencies in this field (PH). [...] in medical school you never really discuss things, but if you do, then only about [...] therapy decisions, but then there is always a right and an okay, second choice. (P5)

VS33	the essential difference is, of course, [...] that in PG and climate change we ultimately discuss [...] issues of existential importance and, of course, the ethical implications are definitely more far-reaching and more difficult to weigh than in a classical natural science subject. (P9)
VS15	It goes far beyond the classical medical curriculum. Also the concept: the planet is sick, let's see what the symptoms are. (P10)
VS116	I don't see any fundamental differences, but perhaps in the sense that one could think differently about social participation or the inclusion of social groups. (P10)
VS117	in any case, it should go beyond the typical medical memorization and crossing off of boxes. (P11)
VS118	And the approach alone, how you study for a degree, [...] how you study for exams in medical school, how you prepare for exams, that's always very to the point, zack, and learn by heart, and zack. You don't really deal much with the topics in detail, discussions are often missing. (P12)
VS119	Good teaching is important in itself, and if you have a new topic, then you can also implement it in good teaching, which means interactively, somehow not only in one direction, not only on knowledge, but also on understanding, and also discuss the topic, and things like that. Develop projects. (P5)
VS120	So in no case lectures [...], from the outside a seminar format and then problem-oriented, interactive, in a lot of small group work, based on concrete examples. (P8)
VS67	the coordination between what you can work on in guided self-study and then implement, apply, discuss, deepen in contact study appropriately prepared. And that is independent of which topic, or whether it is only pure knowledge transfer, or perhaps application knowledge, inclusive practical knowledge, I think you can think this blended learning concept, this distinction between guided self-study and contact study in all areas. (P16)
VS121	I think it's important for me to take a generalist approach and not just think in terms of lectures, because new projects are always just: oh, we'll do a series of lectures on this. But that seminaristic, social, yeah, forms of interaction are also considered. (P16)
VS122	such as on conversation and climate change, so also communication and climate change, how is it communicated in the media, how do we talk about climate change ourselves, do we talk about it at all. (P2)
VS123	a core piece is then also the group work, which is finally then also the achievement proof of the students.(P2)
VS124	And after that, simply, I would say, as an interactive teaching format, in the sense of how do I conduct a climate consultation, in group discussions, that you have actor-patients who you tell, with a good communication strategy, how to change their lifestyle. (P6)
VS125	Of course, you first have to learn a little bit about what PH is, why it's important, that would have to be told to you by someone who knows about it, as a kind of lecture. (P6)

VS126	this... frontline format, if you will. But that's okay, it's embedded in a broader curriculum, where the students then work on it in small groups and create their own posters. (P8)
VS127	But I can very well imagine a blended learning format, where the students first get the information they work with from the typical medical bulimia learning, that's fine by me, but then they should just work with it [...]. I mean, you also need information to be able to discuss. (P8)
VS128	We have already done this together with public health students [...] from different backgrounds. So they had to analyze, for example, some care concepts in the context of prevention and say what is going well and what can be done better. (P10)
VS129	That you create spaces for constructive collective reflection, together with experts on the various topics. (P3)
VS64	that we think more, yes, outside the classical boundaries. Maybe a visit to a farm, how does food production work, what factors play a role. So this is a very fundamental problem that doctors have only a very superficial overview of so far, I think. (P10)
VS130	I think that through this experience, through this practical orientation, you can set the topic, like any other topic in pedagogy of course, much better than if you first pray about it abstractly, theory-led.
VS131	What is always good are these summer schools or winter schools, that you really go out with the students. [...] So this getting out of the normal lecture hall. (P14)
VS132	(an) interactive teaching format, in terms of how do I conduct a climate talk, in group discussions. (P6)
VS133	that they write an essay on a self-determined topic, so they can really choose completely freely, they have to coordinate it with us [...] And based on this, on this scheme, which the students still get, we have simply created an evaluation grid, according to which we can then simply grade the essay. (P1)
VS134	to get the certificate of achievement then for three semester hours, now part of this Planetary Health Academy, since there are lectures. (P8)
VS135	that you analyze situations, perhaps also in teams on site with different thematic focuses from this context PH and exemplarily also in detail really with interviews and impressions in order to then perhaps also make suggestions how you can deal with it better in the future. [...]<br>I imagine such formats to be interesting, that would certainly be something formative and would then not be evaluated with points, but would then also be discussed [...] in a discourse like this [...] and provided with feedback. I could imagine something like that. Of course, it would be time-consuming, but I think it would be interesting. (P10)
VS136	Writing essays, where you really have to think about these things with a written text, so these would all be things that you would have to promote more, which of course takes a lot of time, but I think that's the only way you can actually promote and create a deeper

	understanding. (P12)
VS39	You can't do Global Health anymore without Planetary Thinking. And it makes just as much sense to talk about, I think, Planetary Health if you don't take into account issues of Global Justice and Health Systems and all the classic Global Health issues. (P3)
10. Education as a driver of innovation	
VS137	For me, in the end, good teaching would be a complete mindset change of the teaching staff. So, I would like to see a critical attitude of all university lecturers, be it cardiologists or dermatologists... or pneumologists, who are at the same time also [...] sensitive of the state of our planet and the cross connections to their particular field. And that they would then address this there. (P8)
VS138	I would say that 80 percent of the medical students or my colleagues in the hospital, when I tell them about it, [...] they hear it for the first time that these connections exist. So that is, I think, totally existential. (P12)
VS87	that we also have young people in our studies who we accompany a bit on their life's journey, and with whom we simply have to set the topic. (P13)
VS139	if you take it out of the medical context, where many people at least have a concept of health and understand very quickly why a change has any health effects at all, to make people even more aware of this and thus create a foundation stone. (P15)
VS140	that's also important, that you can pick up the people, so to speak, and deepen it even more and then also empower them, the people, that's super important, but at the same time I think it's just as important and exciting to sensitize people who don't yet have so much to do with it. (P17)
VS141	But at the same time I always had the feeling afterwards: okay, with this [...] I could now move more in a different way than, in quotation marks, "only" with people who are already sensitized. (P17)
VS17	that one simply develops an understanding that the part we call medicine [...] is often a very small part of what actually defines the health of all of us, and above all will also define health in the future. (P11)
VS142	The awareness has to get into the heads even more. Surgeons must understand even more that it is of course important to explain their surgical techniques, but that everything is always connected to everything else, so this idea of Humboldt must radiate into all disciplines. The surgeon must also think outside the box. [...] Of course, this applies at the same time to all other disciplines, internal medicine, ENT and so on and so forth. (P14)

S6: Original German verbatims

1. Complexity and systems thinking	
VS1	Deswegen ist es auch wirklich, finde ich, immer noch eine große Herausforderung, sich da irgendwie ein gutes Programm aufzustellen, wo man einen roten Faden hat und trotzdem irgendwie in die Tiefe geht, weil bisher hatte ich halt auch das Gefühl, dass es dann halt eher an der Oberfläche bleibt. (P1)
VS2	das wird dann irgendwann natürlich auch so breit und so beliebig, dass man hier nur noch sehr schwer überhaupt eine Abgrenzung führen kann. Das ist sicherlich auch eine Gefahr bei dem Ganzen. (P8)
VS3	ich finde, das ist der Reiz, aber auch die Schwierigkeit, dass es eben so breit ist. (P10)
VS4	die aktuelle Situation auch historisch einzuordnen. [...] dass wir wirklich in einer Phase sind, wie ich am Anfang angedeutet habe, wenn wir uns mal zurückversetzen, wie die Entwicklung der modernen Medizin und der modernen Gesellschaft angefangen hat vor 150, 200 Jahren, da hat sich einfach im globalen Norden in der kolonialen Struktur einfach eine Entwicklung durchgesetzt, die wirklich pathogen ist. (P3)
VS5	Themen wie Kommunikation, wie soziale Aspekte, wie Umweltbeziehungen und medizinischen Geografie. (P3)
VS6	Und wir haben das so aufgebaut, dass am Anfang erstmal, also ein gemeinsames Verständnis entwickelt wird von PG und dann im Laufe der Veranstaltung haben wir verschiedene Fokus gelegt, also zum Beispiel welche Zusammenhänge gibt es mit Klimawandel und Gesundheit, welche konkreten Erkrankungen, also zum Beispiel Allergien und wie hängt das zusammen mit dem Klimawandel oder Hitzewellen. (P2)
VS7	vor allem geht es aber auch da wieder um… ja natürlich um Faktenwissen. (P3)
VS8	man muss natürlich erstmal sich ein bisschen aneignen, was ist PG, warum ist es wichtig, das müsste einem einfach jemand erzählen, der davon Ahnung hat, als eine Art Vortrag. (P6)
VS9	Für mich ist es erstmal das grundlegende Verständnis von [...] Klimawandel-Veränderungen und daraus abgeleitet das Verständnis der Auswirkungen auf menschliche Gesundheit. (P16)
VS10	Aber letzten Endes geht es für mich in der PG immer [...] um die Auswirkung auf Patientinnen, Patienten und natürlich auch um den präventiven Charakter, was können wir im Gesundheitswesen tun, um dem Klimawandel Aufschub zu leisten. (P16)
VS11	Ich finde es ganz wichtig, dass man das auch nicht so kürzt auf Umwelt und Gesundheit, sondern dass man ganz viel miteinbezieht, also wie alles zusammenhängt. (P5)
VS12	ich finde es immer in den Vorlesungen, die wir so in der Uni haben, schwierig, wenn man nur einen Aspekt betrachtet oder nur eine Sache

	sich anschaut. Aber es ist ja ein riesiges System, und um das zu verstehen, finde ich, muss alles irgendwie beleuchtet werden und teilweise auch nicht medizinische Sachen. (P5)
VS13	dass man es nicht kürzt und dass man eben sozusagen diese ganze, [...] riesige System, was man ja gar nicht verstehen kann, weil es so groß ist, das aber irgendwie so zulässt. (P5)
VS14	wenn man [...] das System vom Klimawandel verstehen will, [...] dann muss man nicht alle Beispiele abarbeiten, und dann alle Beispiele wissen, sondern da reicht es, ein paar zu bearbeiten und dann hat man einfach ein Verständnis, wie solche Strukturen funktionieren und wie man solche Sachen neu denken kann. (P5)
VS15	es geht ja weit über das medizinische klassische Curriculum hinaus. Auch das Konzept, also: der Planet ist krank, gucken wir mal, was die Symptome so sind. (P10)
VS16	den Studierenden und angehenden Mediziner:innen die Augen dafür zu öffnen, dass die Gesundheit eben nicht an der Krankenhaustür oder an der Praxistür endet, sozusagen, sondern, dass alles, was in unserem Leben und in unserem Alltag passiert, Gesundheit beeinflusst und dass es dann eben auch noch global beeinflusst wird. (P11)
VS17	dass man einfach so ein Verständnis dafür entwickelt, dass der Teil, den wir als Medizin bezeichnen, […] oft ein sehr kleiner Teil ist zu dem, was eigentlich Gesundheit von uns allen ausmacht, und vor allem auch Gesundheit in der Zukunft ausmachen wird. (P11)
VS18	Grundwissen natürlich vermitteln von globalen Klima- und Umweltveränderungen, [...] und dann muss man das eben aber auch schaffen, dass die Studierenden von alleine da draufkommen, [...] diese Querverbindungen zu ziehen. (P12)
VS19	außerhalb von Silos denken. Das Gesamte ansehen [...] den Menschen als Ganzes zu sehen in der Umwelt. Ich glaube, das ist die Kompetenz, die da ganz im Vordergrund steht. (P14)
2. Inter- and transdisciplinarity	
VS20	aber ich finde, gerade bei der Planetaren Gesundheit ist es halt enorm weit aufgefächert und [...] da hatten wir halt auch Referenten aus den verschiedensten Bereichen. (P1)
VS21	es unterscheidet sich vor allem in der Interdisziplinarität, weil, [...] bei der klassischen Lehre, klar, gibt es auch Fächer, die schon sehr interdisziplinär sind, wo wir auch verschiedenste Felder mit einbeziehen, aber ich finde, gerade bei der Planetaren Gesundheit ist es halt enorm weit aufgefächert. (P1)
VS22	man kann nur versuchen die verschiedenen vielfältigen Facetten zu berücksichtigen und darum ist das interdisziplinär und muss einfach auch mit Kolleginnen und Kollegen zusammenarbeiten, die andere Perspektiven und Ideen haben und mit Leuten, die andere Perspektiven und Ideen haben. (P3)

VS23	Zum Beispiel fände ich es cool, wenn wir was mit der Landwirtschaft und auch mit [...] Bauern zusammen machen oder so. [...] Also mit denen, die sich in ihrer Existenz bedroht fühlen. Man muss mit denen zusammen was machen, man hat ja doch eigentlich die gleichen Ziele. (P3)
VS24	wenn eben die Dozierenden aus verschiedensten Disziplinen kommen würden und vielleicht auch die, die Lernenden quasi nicht nur einer Disziplin angehören würden, weil es ja ein überdisziplinäres Thema ist. Und wenn man da wirklich auch aufeinander zukommen möchte und besser Kontakte knüpfen möchte, dann wäre es doch quasi nur logisch, wenn man das dann zusammen lernt in den verschiedenen Disziplinen mit den verschiedenen Disziplinen. (P4)
VS25	Um es zu verstehen, muss man halt auch mal in andere wissenschaftliche Bereiche reinschauen, in soziale Bereiche und kulturelle Dinge. (P5)
VS26	ich fand es total toll, eben mal mit Geographen, Geologen, Lehramtsstudierenden, Psychologen, also da zusammenzuhocken und über Lehrdidaktik zu reden. Und mich hat das sehr bereichert. Und da hatte ich halt auch schon diesen transdisziplinären Gedanken, der ja eben in PH eine enorme Rolle spielt. (P8)
VS27	aber ich habe eher den Eindruck, dass es ein gemeinsames Verständnis unseres Tuns in der Medizin beinhaltet. Über alle Professionen hinweg. (P10)
VS28	Das ist natürlich ein großes Thema, das da Interprofessionalität, Interdisziplinarität betrifft. (P10)
VS29	der Hauptunterschied aus meiner Sicht, ist jetzt die Einbeziehung von anderen, bisher nicht klassisch bei uns mitgedachten Gruppen und Teilhabenden in der Gesellschaft. (P10)
VS30	Gute Lehre in PG ist hoch inter- und transdisziplinär. Weil, man kann nicht eine Vorlesung als Dermatolog:in halten im Bereich PG, sondern man muss da eben mit interdisziplinären Kollegen zusammenarbeiten, oder mit Kollegen aus anderen Disziplinen, das heißt also, die Vorlesung der PG sollte eine interdisziplinäre Vorlesung sein. Aber letztendlich wäre das [...] das Paradebeispiel für eine interdisziplinäre Vorlesung, wo man auch Geographen mitlesen lassen sollte, wo man auch Weltraumforscher lesen lassen sollte und so weiter und so fort. (P14)
VS31	und wenn wir diese interdisziplinäre Lehre, die immer so an das Ende denkt, also an die Prävention nämlich der Menschen, dass die Menschen gesund bleiben am Ende. Dann sollte diese Art der Lehre, die wir für PG wählen, [...] in andere Disziplinen mit reinstrahlen. (P14)
3. Ethical dimension	
VS32	und das Ganze auch ethisch bewerten können. (P9)
VS33	der wesentliche Unterschied ist natürlich, [...] dass wir bei PG und Klimawandel letztendlich [...] Fragestellungen von existenzieller

	Bedeutung diskutieren und natürlich die ethischen Implikationen durchaus weitergehend sind und schwerer zu gewichten sind als in einem klassischen naturwissenschaftlichen Fach. (P9)
VS34	Sondern wir sind bemüht, da auch mal, gerade im deutschen Gesundheitssystem, Dinge zu tun, die schon Ressourcen verbrauchen. Also es ist die Frage, ob eine Chemotherapie, wenn ich die gebe, nicht vielleicht schon eine ganz schlechte CO2- Bilanz hat, kann ich das überhaupt noch wettmachen. Aber, das sind dann ja auch wieder ethische Fragen. (P16)
VS35	Aber während man die Inhalte bearbeitet, dabei so diese grundlegenden Prinzipien aufzugreifen. Wie zum Beispiel soziale Gerechtigkeit. [...] Oder auch das große Thema Gender und Geschlecht, oder auch Rassismus, Sexismus.[...] Und aber auch gleichzeitig eine [...] Kapitalismuskritik ist für mich auch ganz wesentlich. (P17)
VS36	dass man genau überlegt, nach welchen grundlegenden Prinzipien können wir irgendwie unsere Gesellschaft ausrichten. (P17)
VS37	Wie zum Beispiel soziale Ungleichheit und die anderen Sachen, [...] in den ganzen verschiedenen Umwelt-Gesundheits-Wechselwirkungen mit drinstecken und präsent sind. Und, dass das mit drinsteckt, ist für mich das Wesentliche eigentlich. (P17)
VS38	Auch PG bezogen auf die soziale Realität auf der Welt, dass man das auch nicht allein naturwissenschaftlich et cetera sieht, und aus der Brille des Globalen Nordens sondern auch eben die sozialen Fragen, sowohl soziale Ungleichheit bei uns bezogen auf Klimawandel und aber auch Klimafolgen. (P3)
VS39	Man kann Global Health nicht mehr machen ohne Planetary Thinking. Und genauso gut macht es Sinn über, glaube ich, über Planetary Gesundheit zu sprechen, wenn man nicht Fragen von Globaler Gerechtigkeit und Gesundheitssystemen und all die klassischen Global Health Themen mitberücksichtigt. (P3)
VS40	das ist ein Thema glaube ich, zu PG, was bei uns noch nicht so stark betrachtet wird, darum finde ich diese globale und auch die soziale und Fragen von sozialer Gerechtigkeit, globaler Gerechtigkeitsperspektive da ganz wichtig. (P3)
VS41	Ja, vielleicht auch nochmal so ein Verständnis von der Ungleichheit, wie zum Beispiel Klimawandel verschiedene Regionen verschieden trifft. (P4)
VS42	vielleicht auch noch eine Reflexion [...], inwiefern wir noch nicht wirklich decolonized global health haben und sowas. (P4)
VS43	mit so einer Nachhaltigkeitskommission, wo wir wirklich eine Dienstreise- und Reisekonzeption für die Uni ausarbeiten, wo wir auch die Studierenden da mit reinnehmen, um einfach zu überlegen, wann ist es eigentlich noch gerechtfertigt, auch eine Flugreise zu machen. (P3)
VS44	Und Flugreisen komplett zu vermeiden und ansonsten so weiterzuleben wie bisher bringt ja auch nichts, das ist so ein bisschen eine Pseudohaltung: ja ich fliege nicht mehr, ne? Und das ist dann zum Teil auch sehr oberflächlich, ja man muss auch das wieder umfassender, vieldimensionaler denken und wirklich sein eigenes Handeln reflektieren, aber nicht an den Oberflächen bleiben von „ich

	mach das nicht mehr“ oder „[...]niemand darf mehr Fleisch essen”. (P3)
VS45	die Gesundheit so zu denken, dass unser Handeln hier auch Auswirkungen weltweit hat, also, dieses think local, act global. (P2)
VS46	global denken und lokal handeln, wirklich lokal auch Verantwortung empfinden und lokal Initiativen machen. (P3)
4. Responsibility of health professionals	
VS47	kritisches Nachdenken und Reflexion, wo man den eigenen Standpunkt, die eigene Position reflektiert, also die eigene Positioniertheit in unserer Gesellschaft, in der globalen Gesellschaft, bezogen auf all die Fragen, die für PG wichtig sind. (P3)
VS48	wir haben auch einen Beitrag daran und sind auch mit Schuld oder mit dafür verantwortlich oder sind auch in der Position privilegiert, irgendwas daran zu ändern. (P5)
VS49	sie (PHE) muss halt auf jeden Fall den Studierenden klar machen, welchen Stellenwert sie als Ärtz:innen später in ihrem beruflichen Leben für diese Sache spielen können und dass es einen immensen Unterschied macht, ob sie diese Verantwortung wahrnehmen oder eben nicht. (P7)
VS50	eine Hauptkompetenz wäre wirklich, dass Studierende später dann ihr ärztliches Handeln immer wieder kritisch… also die Auswirkungen ihres ärztlichen Handelns kritisch reflektieren können, im Bezug auf Mechanismen, die eben der Gesundheit unseres Planeten entweder dienen oder aber auch im Wege stehen. (P8)
VS51	aber dass ich vielleicht zum Beispiel auch reflektiere überhaupt, zum Beispiel meine ganze Praxisführung, ja, woher beziehe ich meinen Strom, ja, muss ich unbedingt immer mit dem Auto zu Hausbesuchen fahren oder tut es da vielleicht auch ein E-Bike, also dass ich so eine holistische Sichtweise auch auf mein eigenes Handeln habe. (P8)
VS52	Die Ärzte neigen ja häufig dazu, immer andere schlau zu beraten, aber wir können natürlich auch bei uns selber anfangen, was wir selber dazu tun können. (P8)
VS53	dass sie (die Lernenden) ableiten können und diskutieren können, welche Folgen gewisse Handlungen haben, oder auch Nicht-Handlungen haben. (P9)
VS54	wie stark sind wir alle auch Vorbilder in unserem Verhalten als Bürgerinnen und Bürger. (P10)
VS55	finde ich persönlich total wichtig, auch so ein Bewusstsein irgendwie für die gesellschaftliche Verantwortung,[...] die man trägt in diesem Beruf (Gesundheitssektor), dieses [...] Pflichtbewusstsein und Verantwortungsbewusstsein. (P12)
VS56	Was bedeutet das für mein berufliches Ziel, dass ich das anstrebe, was bedeutet es aber auch für mein persönliches Leben? (P13)
VS57	und andererseits aber auch unser eigenes Handeln im medizinischen Sektor hinterfragen und lernen, was unser Handeln und was [...] die Verschreibung von verschiedenen Medikamenten, solche Sachen, für Auswirkungen haben. (P15)

VS58	ich bin mir nicht mal sicher, ob das primär über einen Kompetenzbegriff zu fassen ist, also, ich glaube, dass am ehesten jetzt mit meinem Verständnis von Rollenbildern und Kompetenzen hier so eine Art… Professionalität damit gut verbindbar sind. (P10)
VS59	Insofern glaube ich, dass es eher eine Haltungs- und eine Rahmungsfrage ist, fast eine Kulturfrage. (P10)
VS60	jeder muss davon was gehört haben und ein Verständnis dafür entwickeln. Attitude für alle und Vertiefung für die Interessierten [...] die Medizinstudierenden als kritische Bürger und die Ärztinnen und Ärzte als verantwortungsvolle kritische Bürger im weiteren Sinne. (P10)
5. Transformative competencies including practical focus	
VS61	Und auch so die positiven Sachen dann davon aufzeigen kann, also was passiert, [...] wenn wir das und das ändern, wie wirkt sich das positiv aus. (P5)
VS62	Und dass es auch super motivierend sein kann… im Vergleich zu anderen Fächern (P5)
VS63	gute Lehre ist eben mehr als einfach nur Fakten auswendig zu lernen. Gute Lehre beinhaltet eben insbesondere auch, die Zusammenhänge zu vermitteln und in ein, in ein Handlungswissen zu überführen, sodass Studierende dieses Wissen tatsächlich auch im eigenen Handeln anwenden können, dass sie das Wissen auch reflektieren können, dass sie ableiten können und diskutieren können, welche Folgen gewisse Handlungen haben, oder auch Nicht-Handlungen haben. (P9)
VS64	sondern tatsächlich auch zu sagen, wir bieten da Kochkurse für Medizinstudierende an, zusammen mit einer anderen Profession, machen das praktisch handhabbar und überlegen auch, wie kann denn jemand unter bestimmten sozialen Bedingungen das überhaupt machen. Und gehen vielleicht mal mit Leuten aus der Schule zusammen einkaufen, oder… Also solche Dinge richtig in eine Handlung zu bringen, in eine Handlung die auch jenseits der klassischen Felder, die wir da bespielen, bisher, ist, das fände ich interessant. Also, dass wir da mehr, ja, außerhalb der klassischen Grenzen denken. Vielleicht auch mal ein Besuch bei einem landwirtschaftlichen Betrieb, wie funktioniert denn Erzeugung von Lebensmitteln, welche Faktoren spielen eine Rolle. Also das ist ein ganz fundamentales Problem, das die Ärzte nur sehr oberflächlich, ich glaube, überhaupt bisher überblicken. (P10)
VS65	eine kritische Wahrnehmung, also auch, wie kann ich Dinge, die passieren, kritisch hinterfragen,[...] wie kann ich Dinge, die in der Umwelt oder die global passieren, übersetzen, für mich, für mein tägliches Erleben, für mein tägliches Handeln? [...] und dann aber auch so ein bisschen ein lösungsorientiertes Denken, [...] wie kann man nach vorne arbeiten, was kann auch ich dazu beitragen? (P11)
VS66	da ist es ja erstmal die Grundlage, sozusagen für das Verständnis und dann kann das weitergehen mit Lösungsansätzen, kreativen… ja, transformativen Ideen. (P12)
VS67	die Abstimmung zwischen dem, was kann man im angeleiteten Selbststudium erarbeiten und dann entsprechend vorbereitet im Kontaktstudium umsetzen, anwenden, diskutieren, vertiefen. Und das ist unabhängig davon, welches Thema, oder ob es nur reine

	Wissensvermittlung ist, oder vielleicht Anwendungswissen, inklusives praktisches Wissen, kann man glaube ich, dieses blended learning Konzept, diese Unterscheidung zwischen angeleitetem Selbststudium und Kontaktstudium in allen Bereichen denken. (P16)
VS68	dass die Studierenden so ein bisschen kreativ und auch einfach frei denken, wie wollen wir das haben, ohne die, ja, sag ich jetzt mal, Fesseln von dem, wie es jetzt ist. Um daraus auch die Kraft zu schöpfen: ja, wir können auch die Welt irgendwie so verändern, wie wir es gern wollen. (P17)
VS69	das ist wissenschaftliches Arbeiten und das würde ich halt erwarten, dass man das wirklich aus einer wisschenschaftlichen Perspektive machen, das heißt einfach, dass man sich die Tatsachen genau und kritisch anguckt, auch wenn sie einem nicht passen und verschiedene Perspektiven miteinander in Verbindung bringt und dann versucht, da eine kohärente, plausible Zwischenlösung zu finden. (P3)
VS70	Arzt oder Ärztin, auch in der Rolle als Wissenschaftler:in [...], naturwissenschaftliche Erkenntnisse zu bewerten, richtig bewerten zu können, auch hinsichtlich der Quellen beispielsweise, sie überhaupt zu finden, sie einordnen zu können, zu reflektieren, zu kommunizieren und aus dem Wissen auch eigene Handlungsoptionen ableiten zu können. (P9)
VS71	dass man die Möglichkeit hat, sich intensiver mit einem Thema auseinanderzusetzen, dass man die Möglichkeit hat, auch sich quasi selber zu informieren, das heißt, dass man lernt, wie kann ich Quellen nutzen, wie komme ich zu guten Quellen zu so einem Thema? (P11)
VS72	aber auch die politischen, sozialen, kommunikativen Aspekte. Und das kann man nicht einfach nur Multiple Choice mäßig, also ein paar Faktensachen natürlich, es gibt einfach Tatsachen, die man auch lernen kann, muss man auch einfach draufhaben. Aber wir wissen inzwischen auch, und das zeigt auch die Pandemie, einfach dadurch, dass wir Tatsachen haben und annehmen, wissen wir noch lange nicht, was zu tun ist und wie man das kommuniziert und erreichen auch keine Verhaltens- und politischen Änderungen, da muss man noch andere Dimensionen angehen. (P3)
VS73	dass man vielleicht tatsächlich noch mal diese extracurriculare Idee außerhalb dieser beiden Zonen des Studiums denkt, also dass man wirklich sagt, wir haben die Aufgabe, stärker, unseren Studierenden, in der Gesellschaft sichtbar zu sein. An Schulen, in Alten- und Pflegeheimen [...] Vielleicht auch tatsächlich mal auf der Straße mit Leuten ins Gespräch zu kommen und zu diskutieren,[...] das üben wir ja gar nicht. (P10)
6. Space for reflection and resilience building	
VS74	Gute Lehre macht für mich aus, dass die Studenten begeistert sind, dass die selber auf Ideen kommen, dass die selber entdecken und Gedanken weiterentwickeln dürfen und dass sie selber Lösungen suchen dürfen und, dass sie dabei auch ermutigt werden und dass jetzt nicht ein Weg vorgegeben ist, sondern dass sie Raum haben, Sachen zu entwickeln. (P2)
VS75	dass es nicht was ist, das so verschult sein sollte, sondern wirklich drauf abzielt, ja, ihre eigenen Gedanken sich zu machen und ihr, auch einen Raum bietet, ihr eigenes Verhalten zu reflektieren, und dabei aber auch begleitet werden. Das finde ich, macht die Lehre da aus, also dass sie auch bei den Erkenntnissen, die man da ja unterwegs gewinnt und die ja manchmal auch sehr ernüchternd sind, frustrierend

	oder auch… ja einen auch sehr bewegen können, dass sie da begleitet werden. (P2)
VS76	Und vor allem muss man, glaube ich, die Studierenden ernstnehmen, mit ihren Fragen und Sorgen, Ideen und Vorschlägen. (P3)
VS77	problemorientiertes Lernen, selbst denken, kritisch denken, wo dann eben nicht nur Fakten abgefragt werden, die man auswendig gelernt hat in den Prüfungen, sondern einfach kritisches Denken gefordert wird. (P6)
VS78	dass sie das Wissen auch reflektieren können (P9)
VS79	wie kann ich Dinge, die passieren, kritisch hinterfragen, [...] wie kann ich sie sozusagen übersetzen, also wie kann ich Dinge, die in der Umwelt oder die global passieren, übersetzen, für mich, für mein tägliches Erleben, für mein tägliches Handeln? (P11)
VS80	systemisches Denken, kritisches Denken muss man natürlich irgendwie versuchen zu fördern. Dinge einfach zu hinterfragen und [...] den Horizont irgendwie zu erweitern. (P12)
VS81	in erster Linie geht es für mich darum, wie können die Studierenden und zukünftigen Ärztinnen und Ärzte [...] mit offenen, wachen Augen und einem reflektiven Verständnis in die Interaktion und Behandlung von, mit Patienten gehen. (P16)
VS82	das scheinbar Selbstverständliche zu hinterfragen.[...] Und das einfach… nicht für selbstverständlich zu nehmen, sondern [...] einfach alles zu hinterfragen, egal was es ist, und auch abzuprüfen, entspricht es dem, wie wir eigentlich die Welt gestalten wollen und leben wollen. (P17)
VS83	Dass wir unser Arbeiten, auch an Gesundheit, auch Lehre und sowas, in reiner Form auch als soziale Beziehungen sehen, dass wir mit Menschen zu tun haben, nicht nur mit Objekten, weder mit den Studierenden noch die Leute, mit denen wir arbeiten irgendwo im Ausland, oder wie auch immer, das sind ja alles Menschen [...] Und langefristige soziale Beziehungen sind einfach auch das, was uns trägt und motiviert. (P3)
7. Special role of students	
VS84	die Studierenden da abzuholen, wo sie sich auch gerade befinden. Also anknüpfen an Themen, die sie vielleicht in den Unterricht auch reinwerfen und dann da die Verbindung eben zu schlagen zu PG. (P8)
VS85	ich denke, dass es keinen Sinn macht, das Thema dogmatisch zu behandeln, sondern, dass es zunächst mal so ist, dass ich denke, dass man die Studierenden auch in das Thema reinholen muss. Und das ist der Beginn von einer guten Lehre zu dem Thema, dass es gut begründet, an der Lebenswelt der Studierenden anschließt. (P13)
VS86	gute Lehre in dem Bereich, ja, sollte einerseits an der Lebenswelt der Studierenden andocken, sollte sie hineinholen in das Thema, und sollte dann auch praxisorientiert immer wieder rückgebunden werden. (P13)
VS87	darüber hinaus denke ich aber, und deshalb mache ich mich auch stark dafür, dass es als Querschnittsthema in allen Veranstaltungen

	möglichst präsent ist, dass wir auch junge Menschen im Studium haben, die wir ein Stück auf ihrem Lebensweg begleiten, und bei denen das Thema einfach setzen müssen. (P13)
VS76	Und vor allem muss man, glaube ich, die Studierenden ernstnehmen, mit ihren Fragen und Sorgen, Ideen und Vorschlägen. (P3)
VS88	dass man immer noch mehr Raum gut gebrauchen könnte, wo auch Austausch stattfindet, weil viele (Studierende) in verschiedenen Bereichen eben Vorwissen haben und wir auch voneinander lernen können, also nicht nur die Dozierenden können den Studierenden was beibringen, sondern eben auch andersrum. (P7)
VS89	dass man das mit den Studierenden zusammen vielleicht auch sogar erarbeitet, weil manche natürlich schon weiter sind, viele in unserem Alter und die Jüngeren die beschäftigen sich wahrscheinlich schon viel mehr damit als die Älteren oder die Dozierenden teilweise und die noch mit ins Boot zu holen ist sicherlich auch gut. Weil viele ja auch eine andere Perspektive haben (P12)
VS90	sehr interaktive Formate, wo die Studierenden ein Thema bekommen und sich dann einfach umfassend informieren und das dann präsentieren, weil sie dann meistens zu größeren Experten werden als die Dozierenden. [...] dieser Seminarcharakter, wo der Studierende zum Dozent wird. (P14)
VS91	Das ist sicherlich auch eine Generationenfrage und möglicherweise auch eine Bottom-up-Strategie, die von Studierenden und einigen Interessierten ausgeht. (P16)
8. Need for curricular implementation	
VS92	dass es halt einfach noch nicht so ausgearbeitete Konzepte gibt, die im Curriculum verankert sind und schon langjährig erprobt, wo man sich halt irgendwie gut was abgucken könnte, sondern jeder macht halt schon noch so ein bisschen für sich, was er gerade wichtig sieht. (P1)
VS93	das curriculare Lehrangebot ist noch sehr sparsam, sehr unterbesetzt und das extracurriculare ist, glaube ich, in letzter Zeit riesengroß geworden (P2)
VS94	Ich glaube, dass wir (…) dort deutlich mehr machen müssen, ja, weil ich glaube, viel ist noch nicht so richtig bewusst, [...] wie das eigentlich bei uns in 20, 30 Jahren sein wird. Ich glaube, wir sind in vielen Bereichen in der Gesellschaft nicht darauf vorbereitet, dass wir deutlich höhere Temperaturen haben werden, oder sowas wie Starkregenereignisse haben werden. (P9)
VS95	und von daher sollte dieses Thema einen breiteren [...] Platz einnehmen. [...] entweder als eigenes Veranstaltungsformat oder als longitudinales Curriculum. (P9)
VS96	strukturell wäre curriculäre Lehre extrem angebracht. Und, ganz oft die Verknüpfung herzustellen, zwischen den Dingen, die wir schulmedizinisch lernen, sag ich mal, und was das mit PG zu tun hat. (P15)
VS97	Die sind längst nicht dabei, das flächendeckend curricular zu implementieren, aber es ist wichtig, dass wir hier eine Keimzelle aufbauen

	und dann darüber hinaus auch das Projekt oder die Inhalte transferierbar machen und vielleicht zukünftig dann auch systematisch ausrollen. (P16)
VS98	ich glaube, es ist ganz wichtig, dass es auch curricular angeboten wird. [...] damit auch einfach die Kapazität in so einem beladenen Studium da ist, also in so einem vollen Studium, und das vor allem auch alle Leute erreicht [...], die noch nicht so viel damit zu tun haben. (P17)
VS99	Und das ist für mich auch ein wichtiger Punkt, dass PH genau wie Global Health eben nicht etwas ist, was insgesamt nur dem Medizinstudium hinzugefügt wird als was ganz neues und was von dem Medizinstudium an und für sich ansonsten getrennt wäre und nur Zusatz, Zusatz, Zusatz ist, sondern es ist ganz klar etwas, was im Kern das Medizinstudium berührt und viele Kompetenzen, die für ärztliche Ausbildung und zeitgemäße ärztliche Ausbildung wichtig sind, extrem gut abbildet und da auch einem großen Interesse und einem großen Bedarf von Seiten der Studierenden entspricht. (P3)
VS100	dass das einfach auch an unserer Uni viel mehr repräsentiert wird. (P5)
VS101	dass es ins Curriculum aufgenommen wird als Querschnittsbereich. (P5)
VS102	dass man die PG eben da implementiert wo sie [...] als Fach Wirkung hat, also beispielsweise halt in der Medizin, wo es ja viel darum geht: wie entstehen Erkrankungen, wie kann ich präventiv arbeiten… Ursachensuche. Und da geht es ja sehr viel um Lebensstil, wo die PG ja eigentlich ein sehr zentrales Thema ist, nur leider in der Lehre noch nicht vertreten ist, weil wir momentan leider nur Sekundär- und Tertiärmedizin, so ein bisschen, gelehrt bekommen, aber sehr wenig Präventionsarbeit. (P6)
VS103	für mich wäre PG ähnlich wie Global Health, wenn man so will, so wie auf Englisch ein cross-cutting issue, was praktisch durch den kompletten Lehrplan, durch das komplette Curriculum ziehen sollte. (P8)
VS104	ich bin der Meinung, dass es ohne Zweifel Themen gibt, die für alle im Pflichtcurriculum bezüglich dieser breiter gedachten PG, die da vorkommen müssen. Ich kann mir vorstellen, dass das auch gut als Wahlbereich konzipierbar ist, gerade auch im vielleicht internationalen Kontext in verschiedenen vielleicht aber auch regionalen Kontexten. (P10)
VS105	wenn man das versucht, in möglichst viele Fachbereiche irgendwie, ja, einzubringen, wie es ja auch in dem NKLM jetzt irgendwie versucht wird, diese Querverbindungen halt immer zu ziehen, dass es einfach halt sichtbarer gemacht wird und Leute irgendwie die connection halt immer wieder sozusagen machen müssen, dass das alles, was eigentlich im Kleinen passiert mit was Größerem verbunden ist. (P12)
VS106	dass es darum geht, das erstmal sichtbarer zu machen, das gesamte Fach in einer longitudinalen Ausrichtung, dass sozusagen nicht im Medizinstudium immer nur einzelne Themen aufploppen, hier mal eine Vorlesung dazu ist, da mal was zu Prävention, sondern, dass die aufeinander aufbauen. (P12)
VS107	dass es eigentlich dringend erforderlich ist, dass zum einen alle Studierenden mit dem Thema konfrontiert werden und dass das eben in

	die, ja Standardcurricula, in die Pflichtveranstaltungen mit aufgenommen wird. Und dass es vielleicht zielführender ist, neben einer einführenden Veranstaltung zum Thema, es doch immer wieder anzudocken in allen klinischen und vorklinischen Lehrveranstaltungen. (P13)
VS108	Ideal würde ich mir ein longitudinales Unterrichtskonzept oder ein modulares Konzept vorstellen. [...] natürlich ist der Stundenplan an allen Stellen übervoll. Und neue Elemente bedeuten auch, dass wir Studierende zeitlich belasten, oder eben auch andere bewährte Themen streichen müssen. An dem Punkt sind wir noch nicht, weil dieses Thema sich neu etabliert und wir es momentan als Wahlfach den Studierenden add-on zur Verfügung stellen. (P16)
VS109	ich kann nicht sagen, es passt in das eine Semester und das andere nicht. Weil die Themen so sehr übergeordnet sind. Möglicherweise ist es gut, ein gewisses Grundverständnis für die Problematik schon bereits in der Vorklinik hervorzurufen und dann im klinischen Abschnitt eher die direkten Auswirkungen auf Erkrankungen, Pharmakotherapie et cetera zu besprechen. (P16)
VS110	Weil die Umweltmedizin ist ja auch genau das (wie PG), wir kümmern uns darum, wie Umwelt krankt macht oder Umwelt gesund hält und wie wir am Ende Prävention betreiben können. Das heißt also, wir müssten die Umweltmedizin mehr ins Curriculum reinbringen, die ist viel zu weit draußen und dann sollte es curriculare Lehre sein. Aber die Umweltmedizin kann hier die koordinierende Rolle übernehmen. (P14)
9. Innovative and proven didactic methods	
VS111	das ist auch nicht anders als bei anderer Lehre, natürlich muss es fachlich korrekt und richtig sein, was man vermittelt. (P3)
VS63	gute Lehre ist eben mehr als einfach nur Fakten auswendig zu lernen. Gute Lehre beinhaltet eben insbesondere auch, die Zusammenhänge zu vermitteln und in ein, in ein Handlungswissen zu überführen, sodass Studierende dieses Wissen tatsächlich auch im eigenen Handeln anwenden können, dass sie das Wissen auch reflektieren können, dass sie ableiten können und diskutieren können, welche Folgen gewisse Handlungen haben, oder auch Nicht-Handlungen haben. (P9)
VS112	ich denke, dass die klassische Lehre sowieso ausgedient hat. Wir müssen sowieso insgesamt überall die Lehre revolutionieren. (P14)
VS113	Gute Lehre ist nicht fachspezifisch. Gute Lehre ist, [...] einfach ein didaktisch ausgeklügeltes und abgestimmtes System, das eben blended learning mit inkludiert. Also für mich ist die reine Präsenzlehre spätestens seit Corona überholt, und ich finde, es ist gut, zu unterscheiden, was dient dem Erkenntnisgewinn und der Wissensvermittlung. (P16)
VS114	Dadurch, dass die PG jetzt de novo implementiert wird, glaube ich, muss sie umso mehr noch ausgeklügelte didaktische Konzepte aufweisen. Ich glaub, es wäre jetzt dem Thema nicht gerecht, einen klassischen Weg zu wählen. Sondern, das muss dann hoch innovativ sein, wie auch das Thema an sich. (P16)
VS21	es unterscheidet sich vor allem in der Interdisziplinarität, weil, [...] bei der klassischen Lehre, klar, gibt es auch Fächer, die schon sehr interdisziplinär sind, wo wir auch verschiedenste Felder mit einbeziehen, aber ich finde, gerade bei der Planetaren Gesundheit ist es halt

	enorm weit aufgefächert (P1)
VS72	aber auch die politischen, sozialen, kommunikativen Aspekte. Und das kann man nicht einfach nur Multiple Choice mäßig, also ein paar Faktensachen natürlich, es gibt einfach Tatsachen, die man auch lernen kann, muss man auch einfach draufhaben. Aber wir wissen inzwischen auch, und das zeigt auch die Pandemie, einfach dadurch, dass wir Tatsachen haben und annehmen, wissen wir noch lange nicht, was zu tun ist und wie man das kommuniziert und erreichen auch keine Verhaltens- und politischen Änderungen, da muss man noch andere Dimensionen angehen. (P3)
VS115	ich glaube, dass man halt auch noch mal ganz andere Kompetenzen bei dem Thema (PG) erwerben kann. [...] im Medizinstudium diskutiert man ja eigentlich nie über Sachen, sondern wenn dann nur über [...] Therapieentscheidungen, aber dann gibt es schon auch immer, so ein Richtig und ein na gut, zweite Wahl. (P5)
VS33	der wesentliche Unterschied ist natürlich, [...] dass wir bei PG und Klimawandel letztendlich, ja, Fragestellungen von existenzieller Bedeutung diskutieren und natürlich die ethischen Implikationen durchaus weitergehend sind und schwerer zu gewichten sind als in einem klassischen naturwissenschaftlichen Fach. (P9)
VS15	es geht ja weit über das medizinische klassische Curriculum hinaus. Auch das Konzept, also: der Planet ist krank, gucken wir mal, was die Symptome so sind. (P10)
VS116	ich sehe da keine, keine fundamentalen Unterschiede, vielleicht aber doch in dem Sinne, dass man mehr so eine gesellschaftliche Teilhabe, oder so ne Einbeziehung vielleicht von gesellschaftlichen Gruppen, anders denken könnte. (P10)
VS117	auf jeden Fall sollte es über das medizintypische Auswendiglernen und Ankreuzen hinausgehen. (P11)
VS118	Und alleine schon die Herangehensweise, wie man für ein Studium lernt, [...] wie man im Medizinstudium für Klausuren lernt, wie man sich auf Prüfungen vorbereitet, das ist ja immer sehr auf den Punkt, zack, und auswendig lernen, und zack. Man beschäftigt sich ja nicht wirklich viel mit den Themen im Detail, Diskussionen fehlen häufig. (P12)
VS119	gute Lehre an sich ist ja schon wichtig und wenn man so ein neues Thema hat, dann kann man das ja auch gleich in guter Lehre auch umsetzen, also interaktiv, irgendwie nicht, nicht nur in eine Richtung, nicht nur auf Wissen, sondern auch auf Verstehen, und auch diskutieren zu dem Thema, und solche Sachen auch alles. Projekte entwickeln. (P5)
VS120	Also auf keinen Fall Vorlesungen [...], von dem äußeren ein Seminarformat und dann problemorientiert, interaktiv, in viel Kleingruppenarbeit, aufgehängt an konkreten Beispielen. (P8)
VS67	die Abstimmung zwischen dem, was kann man im angeleiteten Selbststudium erarbeiten und dann entsprechend vorbereitet im Kontaktstudium umsetzen, anwenden, diskutieren, vertiefen. Und das ist unabhängig davon, welches Thema, oder ob es nur reine Wissensvermittlung ist, oder vielleicht Anwendungswissen, inklusives praktisches Wissen, kann man glaube ich, dieses blended learning Konzept, diese Unterscheidung zwischen angeleitetem Selbststudium und Kontaktstudium in allen Bereichen denken. (P16)

VS121	Ich glaube, wichtig für mich ist, auch wieder der generalistische Ansatz, nicht nur in Vorlesungen zu denken, also neue Projekte werden ja immer gleich: ach wir machen mal schön eine Vorlesungsreihe dazu. Sondern dass eben auch seminaristische, soziale, ja, Interaktionsformen mitgedacht werden. (P16)
VS122	wie zum Beispiel zu Gesprächsführung und Klimawandel, also auch Kommunikation und Klimawandel, wie wird das in den Medien kommuniziert, wie sprechen wir selber über den Klimawandel, sprechen wir überhaupt drüber. (P2)
VS123	ein Kernstück ist dann auch noch die Gruppenarbeit, die letztendlich dann auch der Leistungsnachweis der Studenten ist.(P2)
VS124	Und danach halt einfach als, sag ich mal, interaktives Lehrformat, im Sinne von wie führe ich eine Klimasprechstunde, in Gruppendiskussionen, dass man Schauspielpatienten hat, denen man eben erzählt, mit guter Kommunikationsstrategie, wie man Lebensstil ändert. (P6)
VS125	man muss natürlich erstmal sich ein bisschen aneignen, was ist PG, warum ist es wichtig, das müsste einem einfach jemand erzählen, der davon Ahnung hat, als eine Art Vortrag. (P6)
VS126	dieses… Frontline-Format, wenn man so will. Aber das ist okay, das ist so eingebettet halt in ein breiteres Curriculum, wo die Studierenden aber wiederum dann in Kleingruppen das verarbeiten, ihre eigenen Poster erstellen. (P8)
VS127	ich kann mir aber sehr gut ein blended learning Format vorstellen, wo die Studierenden schon erstmal die Informationen, mit denen sie arbeiten, schon erstmal so… über das typische Mediziner-Bulimie-Lernen beziehen, von mir aus, das ist okay, aber dann sollen sie halt damit arbeiten [...]. Ich meine, man braucht ja auch Informationen, um diskutieren zu können. (P8)
VS128	wir haben das auch schon gemacht, mit Public Health Studenten zusammen [...], aus verschiedenen Hintergründen. Also die mussten dann zum Beispiel irgendwelche Versorgungskonzepte im Präventionskontext analysieren und sagen was läuft da gut und was kann man besser machen. (P10)
VS129	Dass man also Räume schafft für das konstruktive gemeinsame Nachdenken, gemeinsam mit Expertinnen und Experten zu den verschiedenen Themenbereichen. (P3)
VS64	dass wir da mehr, ja, außerhalb der klassischen Grenzen denken. Vielleicht auch mal ein Besuch bei einem landwirtschaftlichen Betrieb, wie funktioniert denn Erzeugung von Lebensmitteln, welche Faktoren spielen eine Rolle. Also das ist ein ganz fundamentales Problem, das die Ärzte nur sehr oberflächlich, ich glaube, überhaupt bisher überblicken. (P10)
VS130	ich denke, über diese Erfahrung, über diese Praxisorientierung, kann man das Thema, wie jedes andere Thema auch in der Pädagogik natürlich, viel besser setzen, als wenn man es erstmal abstrakt, theoriegeleitet [...] herbetet.
VS131	was immer wieder gut sind, sind diese Summer Schools oder Winter Schools, dass man wirklich mit den Studierenden auch mal rausgeht. [...] also dieses Raus aus dem normalen Hörsaal. (P14)

VS132	(ein) interaktives Lehrformat, im Sinne von wie führe ich eine Klimasprechstunde, in Gruppendiskussionen. (P6)
VS133	dass man quasi einen Essay zu einem selbst bestimmten Thema schreibt, also die können das wirklich komplett frei wählen, die müssen das dann mit uns absprechen [...] Und da haben wir dann eben darauf aufbauend, auf diesem Schema, was die Studierenden noch kriegen, halt einfach ein Bewertungsraster erstellt, nach dem wir dann das Essay einfach bepunkten können. (P1)
VS134	um den Leistungsnachweis dann für drei Semesterwochenstunden zu kriegen, jetzt einen Teil diese Planetary Health Academy, da gibt es ja Vorlesungen. (P8)
VS135	dass man Situationen, vielleicht auch in Teams vor Ort mit verschiedenen thematischen Schwerpunkten aus diesem Kontext PG analysiert und exemplarisch auch in die Tiefe wirklich mit Interviews und Eindrücken dann versieht, um dann vielleicht auch Vorschläge zu machen, wie man damit besser in Zukunft umgehen kann. [...]<br>solche Formate stelle ich mir interessant vor, das wäre sicher was formatives und würde dann nicht mit Punkten bewertet, sondern in so einem Diskurs dann auch [...] besprochen [...] und mit Feedback versehen werden. Sowas könnte ich mir vorstellen. Das ist natürlich aufwändig, aber, ich glaube, interessant. (P10)
VS136	Essays schreiben, also wo man sich wirklich mal mit einem Text irgendwie über so Sachen auch Gedanken machen muss, also das wären alles so Sachen, die man auch irgendwie mehr fördern müsste, was natürlich viel Zeit in Anspruch nimmt, aber, ich glaube, nur so kann man so ein tieferes Verständnis auch tatsächlich auch fördern und erschaffen. (P12)
VS39	Man kann Global Health nicht mehr machen ohne Planetary Thinking. Und genauso gut macht es Sinn über, glaube ich, über Planetary Gesundheit zu sprechen, wenn man nicht Fragen von Globaler Gerechtigkeit und Gesundheitssystemen und all die klassischen Global Health Themen mitberücksichtigt. (P3)
10. Education as a driver of innovation	
VS137	für mich wäre letztendlich gute Lehre, ein kompletter Mindset-Wandel des Lehrkörpers. Also, ich wünsche mir praktisch eine kritische Einstellung aller Hochschullehrenden, sei das Kardiologen oder Dermatologen… oder Pneumologen, die aber gleichzeitig eben auch [...] einen Sensor haben für den Zustand unseres Planeten und die Querverbindungen zu ihrem jeweiligen Fachgebiet. Und, dass sie das dann dort eben thematisieren würden. (P8)
VS138	ich würde mal sagen, bei 80 Prozent der Medizinstudenten oder auch jetzt Kollegen und Kolleginnen von mir im Krankenhaus, wenn ich denen darüber erzähle, [...] hören [sie] das gefühlt zum ersten Mal, dass es diese Zusammenhänge überhaupt gibt. Also das ist, glaube ich, total existenziell. (P12)
VS87	dass wir auch junge Menschen im Studium haben, die wir ein Stück auf ihrem Lebensweg begleiten, und bei denen das Thema einfach setzen müssen. (P13)

VS139	wenn man das halt aus dem Medizinkontext rausnimmt, wo ja viele wenigstens einen Gesundheitsbegriff haben und sehr schnell verstehen, warum dann eine Veränderung überhaupt gesundheitliche Auswirkungen hat, das überhaupt ins Bewusstsein zu rufen und so einen Baustein zu legen. (P15)
VS140	das ist auch wichtig, dass man da sozusagen die Leute abholt und da noch weiter vertiefen kann und die dann auch empowert, die Leute, das ist super wichtig, aber gleichzeitig find ich es genauso wichtig und spannend, Leute zu sensibilisieren, die noch nicht so viel damit zu tun haben. (P17)
VS141	gleichzeitig hatte ich danach aber auch immer das Gefühl: okay, damit [...] konnte ich jetzt auf eine andere Art und Weise nochmal mehr bewegen als, in Anführungsstrichen, „nur“ mit Leuten, die eh schon sensibilisiert sind. (P17)
VS17	dass man einfach so ein Verständnis dafür entwickelt, dass der Teil, den wir als Medizin bezeichnen, […] oft ein sehr kleiner Teil ist zu dem, was eigentlich Gesundheit von uns allen ausmacht, und vor allem auch Gesundheit in der Zukunft ausmachen wird. (P11)
VS142	Das Bewusstsein muss noch stärker in die Köpfe reinkommen. Der Chirurg muss noch mehr verstehen, dass es natürlich wichtig ist, dass er seine chirurgischen Techniken erklärt, aber, dass alles immer mit allem in Verbindung gesteht, also dieser Humboldt‘sche Gedanke, der muss in alle Disziplinen reinstrahlen. Der Chirurg muss auch über seinen Tellerrand hinausschauen. [...] das gilt natürlich gleichzeitig für alle anderen Disziplinen, Innere, HNO und so weiter und so fort. (P14)
